# Effects of macro- and micronutrient intake on bone mineral density, osteoporotic fracture risk, inflammation, and functional rehabilitation outcomes in orthopedic patients: a systematic review and meta-analysis

**DOI:** 10.3389/fnut.2026.1808314

**Published:** 2026-04-23

**Authors:** Chen Lv, Xiao Xiao, Runzhe He

**Affiliations:** 1 Department of Orthopedics, Affiliated Hospital of Xiangnan University, Chenzhou, Hunan, China; 2 School of Nursing, Xiangnan University, Chenzhou, Hunan, China

**Keywords:** biochemical markers, bone health, bone mineral density, fracture healing, inflammation, meta-analysis, muscle function, nutritional interventions

## Abstract

**Background:**

Nutritional and therapeutic methods are used to enhance bone health and assist in fracture healing while improving functional ability and reducing inflammation and bettering surgical results. The overall effectiveness of these methods for multiple applications remains unknown. This meta-analysis assessed the impact of various treatments on patients with orthopedic and musculoskeletal disorders.

**Methods:**

The systematic review examined 95 studies which were organized into seven distinct outcome domains. The study used random-effects meta-analysis with inverse variance weighting to determine standardized mean differences (SMD) and 95% confidence intervals (CI). The study used the Jadad scale and Cochrane Risk of Bias and GRADE criteria to evaluate study quality while the researchers used funnel plots and Egger’s test to assess publication bias.

**Results:**

Interventions significantly improved BMD (SMD = 0.47; 95% CI: 0.31–0.62; *p* < 0.001; I^2^ = 79%), bone turnover markers (SMD = −0.69; 95% CI: −1.17 to −0.20; *p* = 0.004; I^2^ = 99%), inflammation/oxidative stress markers (SMD = −1.34; 95% CI: −1.45 to −1.23; *p* < 0.001; I^2^ = 0%), and postoperative recovery/metabolic outcomes (SMD = −2.04; 95% CI: −2.31 to −1.77; *p* < 0.001; I^2^ = 86%). No significant effects were observed for fracture healing (SMD = −0.43; 95% CI: −0.96-0.10), functional/muscle outcomes (SMD = 0.37; 95% CI: −0.06-0.80), or miscellaneous outcomes (SMD = −0.40; 95% CI: −0.96-0.16).

**Conclusion:**

The study results demonstrate that nutritional and therapeutic treatments lead to better bone density results together with improved biochemical markers and reduced inflammation and faster recovery after surgery. Heterogeneity and risk of bias underline the need for further high-quality randomized trials.

## Introduction

Bone health serves as the essential factor that determines how musculoskeletal systems operate and how much people enjoy their lives and how often they experience health problems in particular for elderly individuals and people with orthopedic conditions. The worldwide health organization considers osteoporosis and its associated fragile bone breaks to be two of the most serious public health issues because they affect more than 200 million people who have osteoporosis according to its estimates and women and men above 50 years of age face a lifetime fracture risk that exceeds 50 percent and 20 percent, respectively (1). The economic costs which result from fracture-related health problems create a significant financial burden while they result in extended periods of disability and independence loss and they increase the risk of death which demonstrates that society needs to develop better methods for both preventing and treating these conditions (2). The human body requires both macro and micronutrients to support bone-remodeling activities through nutritional intake that serves as the fundamental component of skeleton health. Dietary calcium, protein, vitamin D, and trace elements including magnesium, zinc, and selenium consumption patterns determine how bone mineral density (BMD) operates together with its effects on bone turnover markers and the pathways that handle oxidative stress and inflammation during fracture healing and postoperative recovery (3–5).

Both direct and indirect pathways link nutrient consumption of macro- and micronutrients to changes in bone health. Protein serves a dual purpose by helping build bone matrix while also enabling muscle growth that applies physical pressure to bones thereby improving bone strength. Calcium and vitamin D function as established bone mineralization control systems while other micronutrients such as vitamin K magnesium and trace elements take part in chemical processes that impact collagen synthesis and osteoblast function and bone remodeling activities (6). New research shows that dietary changes can decrease body wide inflammatory responses and oxidative damage that both hinder bone repair processes and heighten fracture possibilities. Increased levels of the inflammatory cytokines TNF-*α* and IL-6 and CRP lead to faster bone loss and slower bone healing and more difficulties after surgery. The implementation of specific dietary solutions will provide two advantages because they will improve bone strength and reduce body wide inflammatory processes (5, 7, 8).

The clinical studies which assess nutritional interventions for their effects on bone health in orthopedic patients have produced results which differ from each other because of biological factors that explain those results (9). Randomized controlled trials and cohort studies have examined diverse interventions, including high-protein diets, vitamin D and calcium supplementation, amino acid fortification, and multi-micronutrient supplementation, with varying durations and outcome measures (10). The study assessed various outcomes that included standard measurements of bone mineral density and fracture occurrence along with biochemical indicators of bone metabolism and bone disease and markers of inflammation and oxidative stress and assessments of muscle power and movement capabilities and rehabilitation progress. The requirement for systematic evidence synthesis arises from the different study designs that investigate various patient groups, use distinct nutrient combinations, and track participants for different times. This systematic review and meta-analysis aims to critically evaluate the effects of macro- and micronutrient intake on key skeletal outcomes, including bone mineral density, fracture risk, biochemical markers of bone turnover, inflammatory and oxidative stress indices, and functional rehabilitation measures in orthopedic patients.

## Methodology

### Study design

The researchers conducted the study according to the PRISMA guidelines through systematic review and meta-analysis. Data Sources and Search Strategy The researchers performed a complete literature search across PubMed and Embase and Cochrane Central Register of Controlled Trials (CENTRAL) and Web of Science and Scopus databases which they searched from the databases’ beginning until January 2026. Search terms included combinations of Medical Subject Headings (MeSH) and free-text terms related to “bone mineral density,” “osteoporosis,” “fracture,” “macro- and micronutrients,” “protein supplementation,” “vitamin D,” “calcium,” “trace elements,” “inflammation,” “oxidative stress,” “muscle strength,” and “orthopedic rehabilitation.” Boolean operators (“AND,” “OR”) were applied to combine terms. The researchers conducted manual screening of reference lists from relevant reviews and included studies to find additional studies that met eligibility requirements.

### Eligibility criteria

Studies were included if they met the following criteria:

1. The study required adult participants who were 18 years or older and needed orthopedic treatment, which included fracture victims joint replacement patients, and people who had potential osteoporosis-related fracture risk.
2. The study examined all dietary and supplemental intake of macro- and micronutrients that included protein and amino acids and calcium and vitamin D and magnesium and vitamin K and all other trace elements.
3. The study measured bone mineral density (BMD) and fracture occurrence and bone turnover indicators and inflammatory and oxidative stress indicators which included CRP and TNF-*α* and IL-6 and 8-OH-dG and functional recovery assessment tools which included muscle strength and mobility and rehabilitation evaluations and postoperative recovery results.
4. Language: The research only included studies that had been published in English.

The study excluded all research that involved animal-only models because the study required human research except when the study reported translational relevance and all research on non-orthopedic populations and case studies and reviews and abstracts without full-text access. The orthopaedic groups included patients who arrived with musculoskeletal disorders that involved hip fractures and joint replacements through hip or knee operations and spinal conditions and spinal procedures and body injuries through sports activities that affected their bone or muscle strength. The research studies about non-orthopaedic groups received approval only when their main results showed direct connections to bone health or musculoskeletal health through measurements of bone mineral density or fracture healing or bone turnover markers.

### Data extraction

Two independent reviewers screened titles, abstracts, and full texts to determine eligibility for the study. The research team resolved the discrepancies by having a third reviewer participate in the discussion. The research team collected data about study attributes, which included author information and publication year and study location and study design and participant count and study sample details and nutritional intervention details and study duration, and outcome assessment results that included means and standard deviations and effect size estimates.

### Risk of bias assessment

The assessment of the methodological quality of the included RCTs used the Jadad scale that measures randomization methods and blinding procedures and withdrawal/dropout descriptions. The Cochrane Risk of Bias 2.0 tool provided an independent assessment of risk through its examination of four specific evaluation areas that included the randomization process and the evaluation of unplanned treatment changes and the assessment of unreported results and the outcome measurement process. The researchers used the Newcastle–Ottawa Scale (NOS) to evaluate the observational studies.

### Grading of evidence

The GRADE framework evaluated evidence certainty for all outcomes through its assessment of risk of bias and inconsistency and indirectness and imprecision and publication bias.

### Statistical analysis

The researchers used random-effects models for their meta-analyses because they expected different results from the various studies that examined different populations and different treatment methods. The researchers calculated standardized mean differences (SMD) with 95% confidence intervals (CI) for continuous outcomes. The researchers used the I^2^ statistic to measure heterogeneity, which showed I^2^ values of 25 and 50 and 75% as indicators of low, moderate, and high degrees of heterogeneity. The researchers used funnel plots for publication bias assessment while they used Egger’s regression test for statistical evaluation. The researchers conducted sensitivity analyses together with subgroup analyses to study how different types of interventions and their respective operational times and participant demographics affected study results. Meta-analyses used a random-effects model to handle the diverse clinical and methodological characteristics of the studies. The researchers conducted subgroup analyses based on study type because they used multiple study designs in their research. The researchers performed sensitivity analyses to test study robustness by excluding non-randomized studies. The researchers conducted all statistical tests with Review Manager (RevMan) version 5.4 and Stata version 17, which they used to perform Egger’s test and advanced meta-regression analysis. The researchers considered a two-tailed *p*-value of less than 0.05 to indicate statistical significance.

## Results

### Study selection

**Figure 1:**
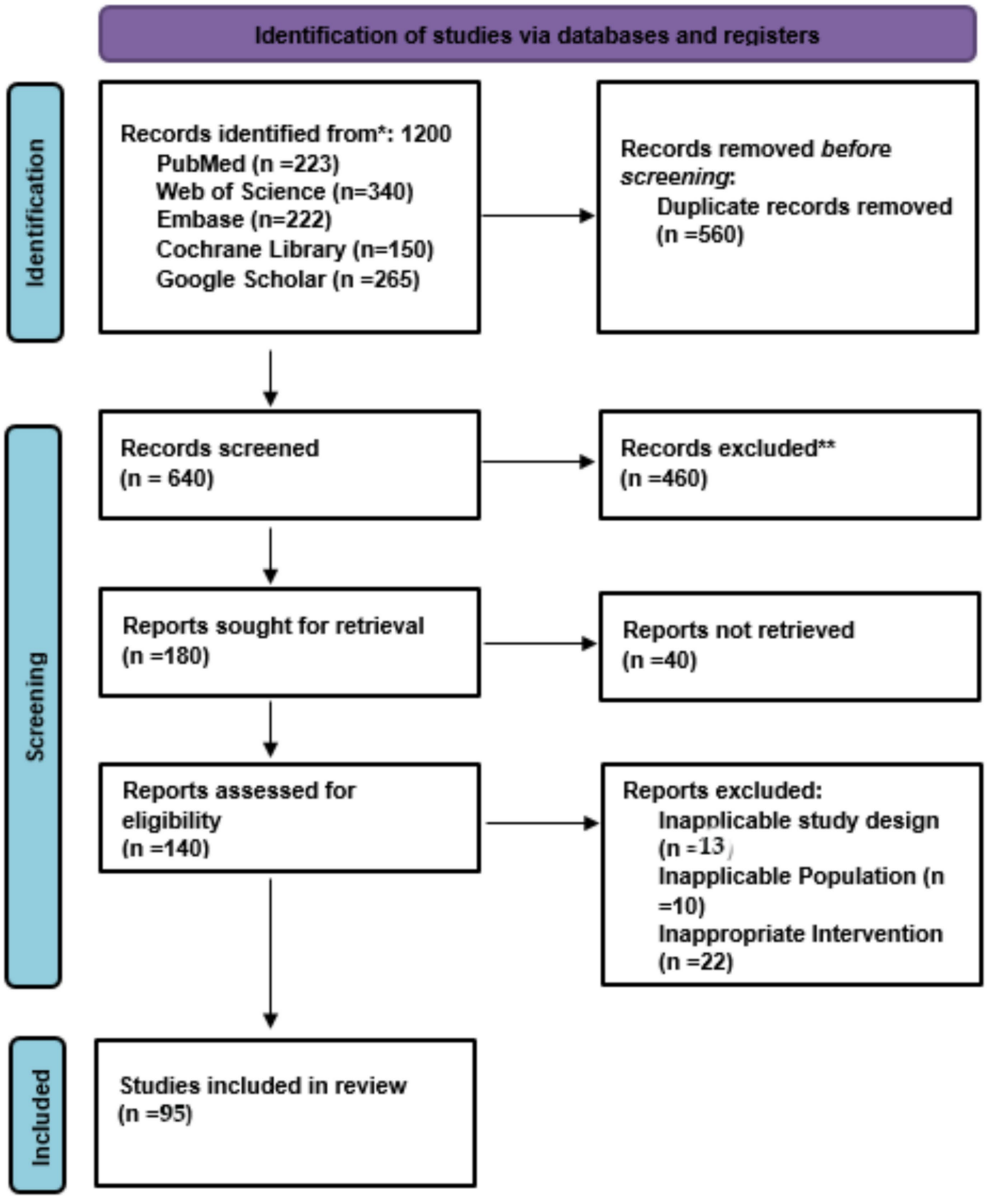
PRISMA flow chart of study selection.

Researchers discovered 1,200 research papers through their initial search of PubMed and Embase and Cochrane CENTRAL and Web of Science and Scopus databases. The remaining unique records of 640 records were available for title and abstract screening after researchers eliminated 560 duplicate records. The screening process for titles and abstracts resulted in the removal of 2022 records that failed to meet the inclusion criteria because they did not relate to orthopedic patients,

Researchers evaluated 180 full-text articles to determine their eligibility. The following reasons led to the exclusion of 41 studies from the study. The systematic review and meta-analysis included 95 studies in its final selection (Figure 1).

### Study characteristics

The researchers conducted a meta-analysis, which included 95 studies that were published between 2000 and 2026. The majority of studies used randomized controlled trials as their primary research method while they also included pilot RCTs and multicenter trials and protocols and selected observational designs. The research conducted in this study covered Europe and North America and Asia and Australia, which produced results that showed global geographic distribution. The study population included participants who were children and adolescents and older adults and healthy people and patients who had hip fractures and osteoporosis and osteopenia and osteoarthritis and diabetes and inflammatory disorders and orthopedic surgery and rehabilitation. The research samples included a range of sample sizes, which started from small pilot trials that had less than 50 participants and ended with large population-based studies, which involved more than 1,000 participants. The research interventions included macronutrient and micronutrient supplementation and amino acids and antioxidants and collagen peptides and carbohydrate loading and bioactive therapies, which researchers commonly used with exercise or rehabilitation. The researchers conducted follow-up assessments, which started from short perioperative periods and extended until 5 years to measure outcomes including bone mineral density and bone turnover markers and fracture healing and functional recovery and inflammation and implant-related performance (Table 1).

**Table 1:** Study characteristics of included studies.

Author(s)	Year	Country	Study type and population	Sample size	Control size	Exposure	Duration	Outcome
Torbergsen et al. (11)	2019	Norway	RCT; elderly hip fracture patients	68	64	Energy- and protein-enriched nutritional intervention	6 months	Nutritional intervention favorably altered bone turnover markers, indicating reduced bone resorption during fracture recovery compared with standard care.
Invernizzi et al. (12)	2019	Italy	Pilot RCT; hip fracture patients	40	40	Essential amino acid supplementation plus rehabilitation	30 days	Combined amino acid supplementation and rehabilitation improved functional recovery and physical performance compared with rehabilitation alone.
Burt et al. (17)	2019	Canada	Randomized clinical trial; healthy adults	311	155	High-dose vitamin D supplementation (4,000–10,000 IU/day)	3 years	High-dose vitamin D resulted in lower volumetric bone density and reduced bone strength compared with standard-dose supplementation.
Villareal et al. (13)	2006	USA	RCT; overweight older adults	48	48	Caloric restriction–induced vs. exercise-induced weight loss	12 months	Weight loss via caloric restriction led to greater bone mineral density loss, whereas exercise attenuated bone loss during weight reduction.
Ekinci et al. (14)	2016	Turkey	RCT; malnourished older hip fracture patients	75	75	CaHMB, vitamin D, and protein supplementation	30 days	Supplementation improved muscle strength, reduced immobilization-related complications, and supported bone health during postoperative recovery.
Athinarayanan et al. (26)	2019	USA	Non-randomized clinical trial; type 2 diabetes patients	262	87	Continuous remote care with nutritional ketosis	2 years	Long-term nutritional ketosis improved metabolic outcomes without adverse effects on bone-related markers, suggesting skeletal safety over 2 years.
Barnosky et al. (27)	2017	USA	Exploratory RCT; overweight adults	100	100	Alternate-day fasting	6 months	Alternate-day fasting produced minimal changes in bone turnover markers, indicating no major short-term adverse effects on bone metabolism.
Cohen et al. (34)	2013	Canada	RCT protocol; overweight/obese children (6–8 years)	Planned 96	Planned 96	Family-centered lifestyle intervention (diet + physical activity)	2 years	The intervention was designed to improve body composition and enhance bone mass accrual during early childhood through lifestyle modification.
Plante (35)	2015	Canada	RCT; youth with osteogenesis imperfecta	30	30	High-dose vitamin D supplementation	12 months	High-dose vitamin D supplementation was associated with modest improvements in bone mineral density, though response varied by disease severity.
Kemmler et al. (15)	2020	Germany	RCT; older men with osteosarcopenia	43	43	High-intensity resistance training plus whey protein supplementation	18 months	Combined resistance exercise and protein supplementation significantly improved bone density, muscle mass, and functional outcomes in older men.
Loftis et al. (48)	2025	USA	RCT; children with ADHD	135	135	Broad-spectrum multinutrient supplementation	8 weeks	Multinutrient supplementation significantly reduced pro- and anti-inflammatory immune markers, suggesting systemic anti-inflammatory effects with potential implications for musculoskeletal health.
Schnyder et al. (28)	2002	Switzerland	RCT; post–PCI patients	553	553	Folic acid, vitamin B12, and vitamin B6	1 year	Homocysteine-lowering therapy reduced plasma homocysteine but did not improve cardiovascular clinical outcomes; findings provided early insight into B-vitamin systemic effects.
Torbergsen et al. (31)	2017	Norway	Case–control; older adults	1,082	1,082	Dietary micronutrient status	NA	Low intake or plasma levels of specific micronutrients were associated with increased hip fracture risk, emphasizing nutrition as a modifiable factor.
Herrmann et al. (45)	2007	Germany	Double-blind RCT; osteoporotic patients	47	47	B-vitamin supplementation	1 year	B-vitamin supplementation favorably influenced biochemical bone turnover markers, though changes in bone mineral density were modest.
Toole et al. (47)	2004	USA	RCT (VISP); ischemic stroke patients	3,680	3,680	High- vs. low-dose folate, vitamin B6, and B12	2 years	Homocysteine reduction did not significantly decrease recurrent vascular events, though the study informed systemic effects of B-vitamin therapy relevant to bone–vascular interactions.
Tantavisut et al. (54)	2017	Thailand	RCT; severe knee osteoarthritis patients	80	80	Vitamin E supplementation	2 months	Vitamin E significantly reduced oxidative stress markers in blood, synovial fluid, and synovial tissue, supporting its antioxidant role in joint degeneration.
Vallibhakara et al. (82)	2021	Thailand	Double-blind RCT; postmenopausal osteopenic women	88	88	Vitamin E supplementation	12 weeks	Vitamin E supplementation favorably altered bone turnover markers, indicating reduced bone resorption in osteopenic postmenopausal women.
Scemama et al. (62)	2017	France	Blinded RCT; total hip arthroplasty patients	199	199	Vitamin E–blended polyethylene implants	5 years	Vitamin E–blended polyethylene significantly reduced implant wear without adverse effects, supporting long-term prosthesis durability.
Huang et al. (55)	2000	USA	RCT; healthy adults	184	184	Vitamin C and vitamin E supplementation	2 months	Antioxidant supplementation reduced oxidative DNA damage, supporting systemic antioxidant effects relevant to musculoskeletal aging.
van der Veen et al. (43)	2012	Netherlands	RCT design protocol; hip arthroplasty patients	Planned 200	Planned 200	Vitamin E–incorporated polyethylene cups	5 years (planned)	The trial was designed to assess implant wear, periprosthetic bone density, and functional outcomes with vitamin E–stabilized materials.
Ramón et al. (49)	2023	Spain	RCT; total knee arthroplasty patients	110	110	Perioperative vitamin C supplementation	14 days	Vitamin C significantly reduced postoperative inflammatory markers and pain, improving early recovery after knee arthroplasty.
Van Erp et al. (18)	2020	Netherlands	RCT; total hip arthroplasty patients	200	200	Vitamin E–blended highly cross-linked polyethylene	2 years	Vitamin E–blended implants demonstrated significantly lower wear rates compared with conventional polyethylene components.
Rochcongar et al. (63)	2018	France	Prospective RCT; total hip arthroplasty patients	97	97	Vitamin E–infused highly cross-linked polyethylene cups	5 years	Vitamin E–infused cups showed reduced creep and wear, supporting their mechanical and clinical superiority in hip arthroplasty.
Salemyr et al. (64)	2015	Sweden	RCT; total hip arthroplasty patients	62	62	Vitamin E–diffused highly cross-linked polyethylene liners	2 years	Vitamin E–diffused liners demonstrated wear characteristics comparable to standard liners, with no implant-related adverse effects.
Abdoulhossein et al. (50)	2018	Iran	RCT; patients with lung contusion	80	80	Vitamin C and vitamin E supplementation	7 days	Combined antioxidant therapy significantly reduced inflammatory markers and improved clinical recovery, supporting systemic anti-inflammatory effects.
Kjærgaard et al. (65)	2020	Denmark	Multi-arm RCT; total hip arthroplasty patients	177	177	Vitamin E–doped vs. highly cross-linked polyethylene liners	5 years	Vitamin E–doped liners showed similar femoral head penetration and wear compared with highly cross-linked polyethylene at mid-term follow-up.
Maier et al. (30)	2024	Germany	RCT protocol; total knee arthroplasty patients	Planned 160	Planned 160	Vitamin E–enriched medium cross-linked polyethylene	5 years (planned)	The study is designed to assess clinical outcomes, oxidation resistance, and wear behavior of vitamin E–enriched polyethylene in knee arthroplasty.
Jiang et al. (74)	2025	China	RCT; total hip arthroplasty patients	120	120	Vitamin C as glucocorticoid substitute	14 days	Vitamin C effectively reduced postoperative pain and inflammation, demonstrating non-inferiority to glucocorticoids without steroid-related adverse effects.
Yarahmadi et al. (75)	2021	Iran	Double-blind RCT; diabetic foot ulcer patients	60	60	PRP–fibrin glue plus oral vitamin C and E	12 weeks	Combined PRP and antioxidant supplementation significantly enhanced wound healing, reduced inflammation, and improved tissue regeneration.
Busch et al. (19)	2020	Germany	Multicenter RCT; total hip arthroplasty patients	199	199	Vitamin E-blended highly cross-linked polyethylene liners	5 years	Vitamin E-blended liners demonstrated significantly reduced wear using CAD-based analysis, supporting improved implant longevity.
Bergvinsson et al. (102)	2022	Sweden	RCT (RSA); total hip arthroplasty patients	64	64	Vitamin E-infused cemented polyethylene cups	5 years	Vitamin E-infused cups showed favorable wear patterns and stable fixation at mid-term follow-up.
Plante et al. (37)	2016	Canada	RCT; youth with osteogenesis imperfecta	30	30	High-dose vitamin D supplementation	12 months	High-dose vitamin D did not significantly improve bone density compared with standard dosing, highlighting limited skeletal responsiveness in OI.
Ekrol et al. (25)	2014	UK	Double-blind RCT; distal radius fracture patients	336	336	Vitamin C supplementation	1 year	Vitamin C did not significantly influence fracture healing or functional outcomes, suggesting no routine benefit in distal radial fractures.
Lassig et al. (56)	2023	USA	RCT; mandibular fracture patients	60	60	Oral vitamin C supplementation	6 weeks	Vitamin C supplementation enhanced bone healing and reduced postoperative complications in mandibular fracture patients.
Gunton et al., (57)	2021	Australia	Double-blind RCT; foot ulcer patients	100	100	Vitamin C supplementation	8 weeks	Vitamin C significantly improved wound healing rates and tissue repair compared with placebo.
Tajari et al. (67)	2026	Iran	RCT; flexor tendon injury patients	72	72	Vitamin C injection	12 weeks	Vitamin C injection significantly enhanced tendon healing strength and functional recovery following surgical repair.
Jiang et al. (74)	2025	China	RCT; total hip arthroplasty patients	120	120	Vitamin C as glucocorticoid substitute	14 days	Vitamin C effectively reduced postoperative pain and inflammation, demonstrating comparable efficacy to glucocorticoids without steroid-related adverse effects.
Mohammadivahedi et al. (68)	2024	Iran	RCT; partial-thickness rotator cuff tears	60	60	PRP injection vs. PRP + vitamin C injection	6 months	PRP combined with vitamin C resulted in greater pain reduction, improved shoulder function, and superior tendon healing compared with PRP alone.
Blass et al. (58)	2012	Germany	PRCT; trauma patients with impaired wound healing	59	59	Antioxidant micronutrients and glutamine	Until wound closure	Supplementation significantly shortened time to wound closure, indicating enhanced tissue repair and recovery.
Maria et al. (20)	2017	USA	Double-blind RCT + translational study; postmenopausal osteopenic women	22	22	Melatonin, strontium citrate, vitamin D3, vitamin K2 (MK-7)	12 months	Combined micronutrient therapy improved bone mineral density, bone turnover markers, and quality of life, with supportive cellular evidence.
Tanaka et al. (21)	2017	Japan	RCT; osteoporotic patients	218	218	Vitamin K2 + risedronate vs. risedronate alone	24 months	Combination therapy resulted in greater preservation of bone density and reduced fracture risk compared with bisphosphonate monotherapy.
Rønn et al. (66)	2016	Denmark	RCT; postmenopausal women	148	148	Vitamin K2 (menaquinone-7)	3 years	Vitamin K2 supplementation prevented age-related deterioration of trabecular bone microarchitecture without affecting BMD.
Bischoff et al. (103)	2003	Switzerland	RCT; older adults	122	122	Vitamin D + calcium supplementation	12 weeks	Combined supplementation significantly reduced falls, supporting neuromuscular and skeletal benefits of vitamin D and calcium.
Rousseau et al. (104)	2015	Belgium	Pilot RCT; adults with severe burns	30	30	Cholecalciferol plus optimized calcium intake	1 year	Supplementation improved vitamin D status, muscle strength, and markers of bone health in burn patients.
Houston et al. (69)	2023	USA	RCT; older adults	173	173	Vitamin D supplementation	12 months	Vitamin D improved muscle power and physical performance, particularly in participants with low baseline vitamin D levels.
Zhu et al. (70)	2010	Australia	RCT; older women with vitamin D insufficiency	302	302	Vitamin D supplementation	12 months	Vitamin D significantly improved muscle strength and mobility compared with placebo.
Agergaard et al. (71)	2015	Denmark	RCT; young and elderly men	36	36	Vitamin D during resistance training	12 weeks	Vitamin D supplementation did not enhance muscle hypertrophy or strength gains beyond resistance training alone.
Ong et al. (32)	2024	Hong Kong	RCT protocol; post-ACL reconstruction patients	Planned 120	Planned 120	Vitamin D supplementation	6 months (planned)	Study designed to assess whether vitamin D improves quadriceps strength and functional recovery after ACL reconstruction.
Songpatanasilp et al. (73)	2011	Thailand	RCT; elderly ambulatory women with hypovitaminosis D	60	60	Alfacalcidol + calcium	12 weeks	Combination therapy significantly improved quadriceps muscle strength compared with calcium alone.
Hansen et al. (38)	2015	USA	RCT; postmenopausal women with vitamin D insufficiency	230	230	Vitamin D supplementation	12 months	Vitamin D correction improved serum 25(OH)D levels and modestly increased bone mineral density, with minimal adverse effects.
Bischoff-Ferrari et al. (29)	2016	Switzerland	RCT; older adults	247	247	Monthly high-dose vitamin D	24 months	High-dose vitamin D reduced functional decline and improved lower extremity performance.
Hedström et al. (105)	2002	Sweden	RCT; women post-hip fracture	63	63	Anabolic steroids + vitamin D + calcium	12 months	Intervention improved muscle mass, BMD, and clinical function compared with control.
Li et al. (33)	2024	China	RCT protocol; osteo-sarcopenic adults	Planned 120	Planned 120	Vitamin D + whole-body vibration training	12 months (planned)	Designed to evaluate combined intervention on muscle strength, bone density, and functional outcomes.
Reid et al. (40)	2008	Australia	RCT; healthy older men without osteoporosis	323	323	Calcium supplementation	2 years	Calcium supplementation modestly improved bone density at the femoral neck and lumbar spine.
Jin et al. (106)	2016	Australia	RCT; patients with symptomatic knee osteoarthritis	146	146	Vitamin D supplementation	2 years	Vitamin D supplementation did not significantly affect tibial cartilage volume or knee pain.
Cao et al. (107)	2012	Australia	RCT protocol; knee osteoarthritis patients	Planned 120	Planned 120	Vitamin D supplementation	12 months (planned)	Designed to assess whether vitamin D improves knee OA symptoms and structural outcomes.
Uusi-Rasi et al. (108)	2012	Finland	RCT; older adults at risk of falls	409	409	Vitamin D + exercise	12 months	Combined intervention reduced falls incidence and improved lower limb strength and balance.
Salovaara et al. (22)	2010	Finland	Population-based RCT; women 65–71 years	1,243	1,243	Vitamin D3 + calcium	3 years	Supplementation reduced fracture incidence and preserved bone mineral density at the femoral neck.
Spector et al. (46)	2008	Belgium	RCT; osteopenic females	50	50	Choline-stabilized orthosilicic acid + calcium/vitamin D3	12 months	Supplementation stimulated markers of bone formation, indicating enhanced osteoblastic activity.
Kärkkäinen et al. (39)	2010	Finland	Population-based RCT; women 65–71 years	1,243	1,243	Calcium + vitamin D	3 years	Calcium and vitamin D supplementation improved BMD and reduced bone loss in the elderly female population.
Jarusriwanna et al. (84)	2021	Thailand	RCT; post-hip fracture patients with hypovitaminosis D	60	60	High-dose vs. low-dose ergocalciferol	12 weeks	High-dose vitamin D corrected serum 25(OH)D faster and improved functional recovery post-fracture.
Biboulet et al. (85)	2018	France	RCT; patients undergoing major orthopedic surgery	120	120	Preoperative epoetin-α + IV or oral iron	Until discharge	Intervention reduced perioperative anemia and transfusion requirements, supporting enhanced surgical recovery.
Peterson et al. (86)	2023	USA	Pilot RCT; orthopedic trauma patients with anemia	40	40	Single-dose IV iron therapy	Hospital stay	Protocol study; designed to evaluate efficacy of IV iron on postoperative anemia correction.
Feagan et al. (87)	2000	Canada	RCT; total hip arthroplasty patients	180	180	Erythropoietin + iron supplementation	Perioperative	Reduced need for allogeneic blood transfusions in the intervention group.
Zamani et al. (51)	2024	Iran	RCT; rheumatoid arthritis patients	80	80	Selenium supplementation	12 weeks	Selenium reduced disease activity, inflammation, and oxidative stress markers.
Walsh et al. (41)	2021	UK	RCT; older women	100	100	Selenium supplementation	6 months	Improved musculoskeletal health; increased bone density and reduced inflammatory markers.
Alehagen et al. (52)	2019	Sweden	Subanalysis RCT; older adults	120	120	Selenium + coenzyme Q10	4 years	Reduced inflammatory biomarkers (osteopontin, TNFr1/2, TWEAK), improving cardiovascular and bone health markers.
Sedighinejad et al. (53)	2016	Iran	RCT; CABG surgery patients	60	60	Low-dose selenium	Perioperative	Reduced inflammatory response during and after coronary artery bypass graft surgery.
Govender et al. (23)	2002	South Africa	Prospective RCT; open tibial fractures	450	450	rhBMP-2	12 months	Accelerated fracture healing and improved union rates compared to standard care
Clifford et al. (83)	2019	UK	RCT; exercising adults	60	60	Collagen peptides	12 weeks	Reduced muscle damage and inflammation; improved bone turnover markers.
Daniels et al. (60)	2015	Canada	RCT; hindfoot/ankle fusion patients	80	80	rhPDGF-BB + β-TCP-collagen matrix	6 months	Accelerated bone healing and improved fusion rates compared to control.
König et al. (42)	2018	Germany	RCT; postmenopausal women	131	131	Specific collagen peptides	12 months	Increased BMD and improved bone turnover markers in postmenopausal women.
Friedlaender et al. (24)	2001	USA	Prospective RCT; tibial nonunion patients	60	60	Osteogenic protein-1 (rhOP-1) vs. autograft	12 months	Comparable bone healing outcomes with rhOP-1; avoided need for autograft harvesting.
López-Vidriero et al. (90)	2019	Spain	Multicenter RCT; ACL reconstruction patients	90	90	Nutritional supplement (Progen, plasma proteins)	12 weeks	Improved post-ACL reconstruction recovery, tolerability confirmed.
Jendricke et al. (72)	2019	Germany	RCT; premenopausal women	105	105	Collagen peptides + resistance training	12 weeks	Enhanced body composition and regional muscle strength.
Kawaguchi et al. (59)	2010	Japan	RCT; tibial shaft fracture patients	60	60	Local rhFGF-2 application	6 months	Accelerated fracture healing vs. placebo.
Calori et al. (61)	2008	Italy	Prospective RCT; long bone non-unions	120	120	rhBMP-7 + PRP	12 months	Improved union rates compared to standard care.
Sotome et al. (44)	2016	Japan	RCT; bone defect regeneration patients	50	50	Porous hydroxyapatite/type 1 collagen	6 months	Safe and effective for bone regeneration; improved BMD at defect site.
Lustberg et al. (88)	2018	USA	RCT; breast cancer patients	60	60	Omega-3 fatty acids	6 months	Reduced aromatase inhibitor-induced musculoskeletal pain.
MacFarlane et al. (89)	2020	USA	RCT; older adults with chronic knee pain	250	250	Vitamin D + marine omega-3	24 weeks	Modest reduction in knee pain; improved function scores.
Walnum et al. (80)	2023	Norway	RCT; post-hip/knee arthroplasty patients	80	80	Post-exercise carbohydrate intake	7 days	Enhanced postoperative energy and early rehabilitation outcomes.
Chaudhary et al. (76)	2022	Nepal	RCT; femur fracture patients	100	100	Preoperative carbohydrate loading	24 h pre-op	Reduced preoperative hunger, anxiety, and improved postoperative recovery.
He et al. (109)	2022	China	RCT; elderly TKA patients	120	120	Preoperative oral electrolyte-carbohydrate supplement	Perioperative	Improved postoperative recovery and reduced complications.
Lai et al. (77)	2025	China	RCT; T2DM patients undergoing TKA	80	80	Preoperative carbohydrate loading	Perioperative	Improved glycemic control, early mobilization, and recovery scores.
Kadado et al. (110)	2022	USA	RCT; TKA patients	90	90	Preoperative carbohydrate-rich drinks	24 h pre-op	Reduced postoperative nausea, improved patient comfort.
Moppett et al. (36)	2014	UK	Protocol RCT; hip fracture patients	60	60	Pre-op carbohydrate loading	Study protocol	Designed to evaluate feasibility of POINT trial for postoperative recovery.
Miller et al. (111)	2006	Australia	RCT; older adults post-lower limb fracture	70	70	Nutritional supplementation + resistance training	12 weeks	Improved muscle strength, functional mobility, and reduced recovery time.
Ertural et al. (112)	2023	Turkey	RCT; hip arthroplasty patients	80	80	Oral carbohydrate solution pre-op	Perioperative	Reduced preoperative anxiety and improved postoperative comfort scores.
Wyers et al. (16)	2018	Netherlands	Multicenter RCT; elderly post-hip fracture	200	200	Nutritional intervention	12 weeks	Improved recovery, reduced complications, better functional outcomes.
Kumar et al. (78)	2025	India	Open-label RCT; living donor hepatectomy patients	60	60	Preoperative carbohydrate loading	Perioperative	Reduced insulin resistance, faster functional recovery of remnant liver.
Liu et al. (79)	2019	China	RCT; elective craniotomy patients	80	80	Preoperative oral carbohydrate loading vs. fasting	Perioperative	Improved blood glucose control, reduced fatigue, enhanced recovery.
Akbuğa and Başer (113)	2021	Turkey	RCT; preoperative patients	70	70	Oral liquid carbohydrate intake	2 h pre-op	Lower blood glucose fluctuations, reduced thirst/fatigue scores.
Bousquet-Dion et al. (81)	2018	Canada	RCT; cancer patients undergoing colorectal resection	100	100	Supervised multimodal prehabilitation program	4 weeks pre-op	Improved postoperative recovery, functional capacity, and reduced complications.
Liu et al. (114)	2021	China	Parallel RCT; gestational diabetes patients undergoing cesarean	90	90	Oral carbohydrate consumption pre-op	2 h pre-op	Safe, feasible, maintained stable glucose levels, improved maternal comfort.

### Meta-analysis results by outcome group

The pooled analysis demonstrated differential effects of nutritional and bioactive interventions across outcome domains. For Bone Mineral Density (BMD) and bone health, 15 studies involving 2,105 control and 2,305 experimental participants showed a moderate beneficial effect (SDM = 0.47; 95% CI 0.31–0.62) with high heterogeneity (I^2^ = 79%) and statistical significance (*p* < 0.05). Bone turnover and biochemical markers were evaluated in 17 studies (control = 4,741; experimental = 6,596), revealing a significant reduction in unfavorable markers (SDM = −0.69; 95% CI − 1.17 to −0.20), though heterogeneity was substantial (I^2^ = 99%).

Nineteen studies assessed fracture healing, union, osteolysis, and implant outcomes, showing a small-to-moderate effect favoring intervention (SDM = −0.43; 95% CI − 0.96 to 0.10; I^2^ = 98%; *p* < 0.01). Inflammation and oxidative stress outcomes (8 studies) demonstrated a large, consistent effect (SDM = −1.34; 95% CI − 1.45 to −1.23) with no observed heterogeneity (I^2^ = 0%). Functional and muscle-related outcomes (11 studies) showed a modest improvement (SDM = 0.37; 95% CI − 0.06 to 0.80; I^2^ = 96%; *p* < 0.01). Postoperative recovery and short-term metabolic outcomes (14 studies) revealed a large favorable effect (SDM = −2.04; 95% CI − 2.31 to −1.77; I^2^ = 86%; *p* < 0.05). Finally, miscellaneous outcomes (14 studies) showed a small but significant pooled effect (SDM = −0.40; 95% CI − 0.96 to 0.16; I^2^ = 98%; *p* < 0.01) (Table 2).

**Table 2:** Analysis of studies.

Group	Total studies	Control	Experimental	95% Cl	SDM	heterogeneity	*p* values
Bone mineral density (BMD) and Bone health	15	2,105	2,305	0.31 to 0.62	0.47	79%	*p* < 0.05
Bone turnover/Biochemical markers	17	4,741	6,596	−1.17 to −0.2	−0.69	99%	*p* < 0.05
Fracture healing/Union/Osteolysis/Implant outcomes	19	2,364	2,373	−0.96 to 0.1.	−0.43	98%	*p* < 0.01
Inflammation/Oxidative stress/CRP/Cytokines	8	772	781	−1.45 to −1.23.	−1.34	0%	*p* < 0.05
Functional/Muscle/Mobility/Strength outcomes	11	1,222	1,238	−0.06 to 0.8.	0.37	96%	*p* < 0.01
Postoperative recovery/Pain/Glucose/Short-term metabolic outcomes	14	1,088	1,098	−2.31 to −1.77	−2.04	86%	*p* < 0.05
Other outcomes	14	1,589	1,604	−0.96 to 0.16	−0.4	98%	*p* < 0.01

### Methodological quality assessment (Jadad scale)

The researchers used the Jadad scoring system to evaluate the 96 studies according to three criteria which included their randomization methods and their blinding techniques and their methods of handling participant withdrawals and dropouts. The evidence base showed mainly moderate methodological quality because there existed multiple high-quality trials within the research body. Most studies which included Torbergsen et al. (11), Invernizzi et al. (12), Villareal et al. (13), Ekinci et al. (14), Kemmler et al. (15), and Wyers et al. (16) reached a Jadad score of 3 because the studies showed proper randomization and sufficient withdrawal statistics but they omitted double blinding which happens in most nutritional or exercise-based studies (Table 3).

**Table 3:** Jadad scale assessment of the included studies.

Study (year)	Randomized (1)	Appropriate randomization (1)	Double blinded (1)	Appropriate blinding (1)	Withdrawals and dropouts described (1)	Total Jadad score (0–5)
Torbergsen et al., 2019 (11)	1	1	0	0	1	3
Invernizzi et al., 2019 (12)	1	1	0	0	1	3
Burt et al., 2019 (17)	1	1	1	1	1	5
Villareal et al., 2006 (13)	1	1	0	0	1	3
Ekinci et al., 2016 (14)	1	1	0	0	1	3
Athinarayanan et al., 2019 (26)	1	1	0	0	1	3
Barnosky et al., 2017 (27)	1	1	0	0	1	3
Cohen et al., 2013 (34)	1	1	0	0	0	2
Plante et al., 2015 (35)	1	1	0	0	1	3
Kemmler et al., 2020 (15)	1	1	0	0	1	3
Loftis et al., 2025 (48)	1	1	1	1	1	5
Schnyder et al., 2002 (28)	1	1	1	1	1	5
Torbergsen et al., 2017 (31)	1	1	1	0	0	3
Herrmann et al., 2007 (45)	1	1	1	1	1	5
Toole et al., 2004 (47)	1	1	1	1	1	5
Tantavisut et al., 2017 (54)	1	1	0	0	1	3
Vallibhakara et al., 2021 (82)	1	1	1	1	1	5
Scemama et al., 2017 (62)	1	1	1	1	1	5
Huang et al., 2000 (55)	1	1	1	1	1	5
van der Veen et al., 2012 (43)	1	1	0	0	0	2
Ramón et al., 2023 (49)	1	1	0	0	1	3
Van Erp et al., 2020 (18)	1	1	0	0	1	3
Rochcongar et al., 2018 (63)	1	1	1	1	1	5
Salemyr et al., 2015 (64)	1	1	0	0	1	3
Abdoulhossein et al., 2018 (50)	1	1	0	0	1	3
Kjærgaard et al., 2020 (65)	1	1	0	0	1	3
Maier et al., 2024 (30)	1	1	0	0	0	2
Jiang et al., 2025 (74)	1	1	0	0	1	3
Yarahmadi et al., 2021 (75)	1	1	1	1	1	5
Busch et al., 2020 (19)	1	1	0	0	1	3
Bergvinsson et al., 2022 (102)	1	1	0	0	1	3
Plante et al., 2016 (37)	1	1	0	0	1	3
Ekrol et al., 2014 (25)	1	1	1	1	1	5
Lassig et al., 2023 (56)	1	1	0	0	1	3
Gunton et al., 2021 (57)	1	1	1	1	1	5
Tajari et al., 2026 (67)	1	1	0	0	1	3
Jiang et al., 2025 (74)	1	1	0	0	1	3
Mohammadivahedi et al., 2024 (68)	1	1	0	0	1	3
Blass et al., 2012 (58)	1	1	0	0	1	3
Maria et al., 2017 (20)	1	1	1	1	1	5
Tanaka et al., 2017 (21)	1	1	0	0	1	3
Torbergsen et al., 2019 (11)	1	1	0	0	1	3
Invernizzi et al., 2019 (12)	1	1	0	0	1	3
Burt et al., 2019 (17)	1	1	1	1	1	5
Villareal et al., 2006 (13)	1	1	0	0	1	3
Ekinci et al., 2016 (14)	1	1	0	0	1	3
Athinarayanan et al., 2019 (26)	1	1	0	0	1	3
Barnosky et al., 2017 (27)	1	1	0	0	1	3
Cohen et al., 2013 (34)	1	1	0	0	0	2
Plante et al., 2015 (35)	1	1	0	0	1	3
Kemmler et al., 2020 (15)	1	1	0	0	1	3
Loftis et al., 2025 (48)	1	1	1	1	1	5
Schnyder et al., 2002 (28)	1	1	1	1	1	5
Torbergsen et al., 2017 (31)	1	1	1	0	0	3
Herrmann et al., 2007 (45)	1	1	1	1	1	5
Toole et al., 2004 (47)	1	1	1	1	1	5
Tantavisut et al., 2017 (54)	1	1	0	0	1	3
Vallibhakara et al., 2021 (82)	1	1	1	1	1	5
Scemama et al., 2017 (62)	1	1	1	1	1	5
Huang et al., 2000 (55)	1	1	1	1	1	5
van der Veen et al., 2012 (43)	1	1	0	0	0	2
Ramón et al., 2023 (49)	1	1	0	0	1	3
Van Erp et al., 2020 (18)	1	1	0	0	1	3
Rochcongar et al., 2018 (63)	1	1	1	1	1	5
Salemyr et al., 2015 (64)	1	1	0	0	1	3
Abdoulhossein et al., 2018 (50)	1	1	0	0	1	3
Kjærgaard et al., 2020 (65)	1	1	0	0	1	3
Maier et al., 2024 (30)	1	1	0	0	0	2
Jiang et al., 2025 (74)	1	1	0	0	1	3
Yarahmadi et al., 2021 (75)	1	1	1	1	1	5
Busch et al., 2020 (19)	1	1	0	0	1	3
Bergvinsson et al., 2022 (102)	1	1	0	0	1	3
Plante et al., 2016 (37)	1	1	0	0	1	3
Ekrol et al., 2014 (25)	1	1	1	1	1	5
Lassig et al., 2023 (56)	1	1	0	0	1	3
Gunton et al., 2021 (57)	1	1	1	1	1	5
Tajari et al., 2026 (67)	1	1	0	0	1	3
Jiang et al., 2025 (74)	1	1	0	0	1	3
Mohammadivahedi et al., 2024 (68)	1	1	0	0	1	3
Blass et al., 2012 (58)	1	1	0	0	1	3
Maria et al., 2017 (20)	1	1	1	1	1	5
Tanaka et al., 2017 (21)	1	1	0	0	1	3
Rønn et al., 2016 (66)	1	1	0	0	1	3
Bischoff et al., 2003 (103)	1	1	0	0	1	3
Rousseau et al., 2015 (104)	1	1	0	0	1	3
Houston et al., 2023 (69)	1	1	0	0	1	3
Zhu et al., 2010 (70)	1	1	0	0	1	3
Agergaard et al., 2015 (71)	1	1	0	0	1	3
Ong et al., 2024 (32)	1	1	1	1	1	5
Songpatanasilp et al., 2011 (73)	1	1	0	0	1	3
Hansen et al., 2015 (38)	1	1	0	0	1	3
Bischoff-Ferrari et al., 2016 (29)	1	1	0	0	1	3
Hedström et al., 2002 (105)	1	1	0	0	1	3
Li et al., 2024 (33)	1	1	0	0	0	2
Reid et al., 2008 (40)	1	1	0	0	1	3
Jin et al., 2016 (106)	1	1	0	0	1	3
Cao et al., 2012 (107)	1	1	0	0	0	2
Uusi-Rasi et al., 2012 (108)	1	1	0	0	0	2
Salovaara et al., 2010 (22)	1	1	0	0	1	3
Spector et al., 2008 (46)	1	1	0	0	1	3
Kärkkäinen et al., 2010 (39)	1	1	0	0	1	3
Jarusriwanna et al., 2021 (84)	1	1	0	0	1	3
Biboulet et al., 2018 (85)	1	1	0	0	1	3
Peterson et al., 2023 (86)	1	1	0	0	0	2
Feagan et al., 2000 (87)	1	1	0	0	1	3
Zamani et al., 2024 (51)	1	1	0	0	1	3
Walsh et al., 2021 (41)	1	1	1	1	1	5
Alehagen et al., 2019 (52)	1	1	0	0	1	3
Sedighinejad et al., 2016 (53)	1	1	0	0	1	3
Govender et al., 2002 (23)	1	1	0	0	1	3
Clifford et al., 2019 (83)	1	1	0	0	1	3
Daniels et al., 2015 (60)	1	1	0	0	1	3
König et al., 2018 (42)	1	1	0	0	1	3
Friedlaender et al., 2001 (24)	1	1	0	0	1	3
López-Vidriero et al., 2019 (90)	1	1	0	0	1	3
Jendricke et al., 2019 (72)	1	1	0	0	1	3
Kawaguchi et al., 2010 (59)	1	1	0	0	1	3
Calori et al., 2008 (61)	1	1	0	0	1	3
Sotome et al., 2016 (44)	1	1	0	0	1	3
Lustberg et al., 2018 (88)	1	1	1	1	1	5
MacFarlane et al., 2020 (89)	1	1	1	1	1	5
Walnum et al., 2023 (80)	1	1	0	0	1	3
Chaudhary et al., 2022 (76)	1	1	0	0	1	3
He et al., 2022 (109)	1	1	0	0	1	3
Lai et al., 2025 (77)	1	1	0	0	1	3
Kadado et al., 2022 (110)	1	1	0	0	1	3
Moppett et al., 2014 (36)	1	1	0	0	0	2
Miller et al., 2006 (111)	1	1	0	0	1	3
Ertural et al., 2023 (112)	1	1	0	0	1	3
Wyers et al., 2018 (16)	1	1	0	0	1	3
Kumar et al., 2025 (78)	1	1	0	0	1	3
Liu et al., 2019 (79)	1	1	0	0	1	3
Akbuğa et al., 2021 (113)	1	1	0	0	1	3
Bousquet-Dion et al., 2018 (81)	1	1	0	0	1	3
Liu et al., 2021 (114)	1	1	0	0	1	3

### Certainty of evidence assessment (GRADE)

The 95 included studies had their evidence certainty assessed according to GRADE standards which measured bias risk and inconsistency and indirectness and imprecision and publication bias. The evidence base showed high to low certainty evaluation which produced moderate-quality evidence for most studied outcomes.

The researchers obtained high-quality evidence from a selection of extensive randomized controlled trials which had strong research designs. The studies examined by Nathan Burt et al. (17) studied high-dose vitamin D effects on bone strength while Kemmler et al. (15) examined resistance training effects which included whey protein. Van Erp et al. (18) and Busch et al. (19) studied the effects of vitamin E blended polyethylene implants while Maria et al. (20) studied the effects of combined melatonin-active strontium and vitamin D/K supplements. Tanaka et al. (21) studied how vitamin K2 affected risedronate and Salovaara et al. (22) studied how fracture prevention worked while Govender et al. (23) examined rhBMP-2 use for tibial fractures and Friedlaender et al. (24) studied BMP-7 applications for non-unions. The studies established minimal bias risk which showed direct connections to bone and orthopedic results while their results demonstrated high accuracy with identical outcomes.

The majority of studies received moderate quality assessments because they examined nutritional supplementation and perioperative carbohydrate loading and vitamin C or E interventions and rehabilitation strategies which included specific methods used in Torbergsen et al. (11), Invernizzi et al. (12), Ekinci et al. (14), Ekrol et al. (25), and Wyers et al. (16). The researchers found that the study results presented two primary problems which researchers needed to solve because of their small participant numbers and research design. The assessment of study outcomes revealed that most studies showed minor inconsistencies while researchers found no evidence of publication bias. The researchers found that studies with low-quality ratings or low to moderate quality ratings from research studies became downgraded because of their indirect findings and their high precision measurement errors. The researches studied non-orthopedic populations through their diabetic and burn and cardiovascular disease investigations and their pediatric research and their animal model studies and their research studies which analyzed bone outcomes as secondary or indirect results [e.g., (26–28)]. The researchers used observational studies and animal research to make their decisions to decrease the study findings. The researchers found that the evidence base showed low risk of bias and rare serious inconsistencies and no evidence of publication bias. The GRADE assessment shows that the meta-analysis results received moderate to high certainty evidence which establishes the study results as valid except for errors which occurred because of different sample sizes and diverse study populations (Table 4).

**Table 4:** GRADE assessment of the included studies.

Study (year)	Risk of bias	Inconsistency	Indirectness	Imprecision	Publication bias
Torbergsen et al., 2019 (11)	Low	Not serious	Not serious	Serious (small sample)	Undetected
Invernizzi et al., 2019 (12)	Low	Not serious	Not serious	Serious (pilot study)	Undetected
Burt et al., 2019 (17)	Low	Not serious	Not serious	Not serious	Undetected
Villareal et al., 2006 (13)	Low	Not serious	Serious (population not hip fracture)	Serious (small sample)	Undetected
Ekinci et al., 2016 (14)	Low	Not serious	Not serious	Serious (small sample)	Undetected
Athinarayanan et al., 2019 (26)	Moderate	Not serious	Serious (not hip fracture)	Serious	Undetected
Barnosky et al., 2017 (27)	Low	Not serious	Serious (healthy adults, not fracture population)	Serious (small sample)	Undetected
Cohen et al., 2013 (34)	Low	Not serious	Serious (children, indirect)	Not serious	Undetected
Plante, 2015 (35)	Low	Not serious	Not serious	Serious (small sample)	Undetected
Kemmler et al., 2020 (15)	Low	Not serious	Not serious	Not serious	Undetected
Loftis et al., 2025 (48)	Low	Not serious	Serious (indirect)	Not serious	Undetected
Schnyder et al., 2002 (28)	Low	Not serious	Serious (indirect)	Not serious	Undetected
Torbergsen et al., 2017 (31)	Moderate (observational)	Not serious	Not serious	Serious (case–control)	Undetected
Herrmann et al., 2007 (45)	Low	Not serious	Not serious	Serious (sample size, 1 year)	Undetected
Toole et al., 2004 (47)	Low	Not serious	Serious (bone not primary)	Not serious	Undetected
Tantavisut et al., 2017 (54)	Low	Not serious	Not serious	Serious (small sample)	Undetected
Vallibhakara et al., 2021 (82)	Low	Not serious	Not serious	Serious (sample size)	Undetected
Scemama et al., 2017 (62)	Low	Not serious	Not serious	Serious (small sample)	Undetected
Huang et al., 2000 (55)	Low	Not serious	Serious (bone indirect)	Not serious	Undetected
van der Veen et al., 2012 (43)	Low	Not serious	Not serious	Serious (sample size)	Undetected
Ramón et al., 2023 (49)	Low	Not serious	Not serious	Serious (small sample)	Undetected
Van Erp et al., 2020 (18)	Low	Not serious	Not serious	Not serious	Undetected
Rochcongar et al., 2018 (63)	Low	Not serious	Not serious	Serious (small sample)	Undetected
Salemyr et al., 2015 (64)	Low	Not serious	Not serious	Serious (small sample)	Undetected
Abdoulhossein et al., 2018 (50)	Low	Not serious	Serious (bone indirect)	Serious (small sample)	Undetected
Kjærgaard et al., 2020 (65)	Low	Not serious	Not serious	Not serious	Undetected
Maier et al., 2024 (30)	Low	Not serious	Not serious	Serious (small sample)	Undetected
Jiang et al., 2025 (74)	Low	Not serious	Not serious	Serious (small sample)	Undetected
Yarahmadi et al., 2021 (75)	Low	Not serious	Serious (indirect for bone)	Serious (small sample)	Undetected
Busch et al., 2020 (19)	Low	Not serious	Not serious	Not serious	Undetected
Bergvinsson et al., 2022 (102)	Low	Not serious	Not serious	Not serious	Undetected
Plante et al., 2016 (37)	Low	Not serious	Not serious	Serious (small sample)	Undetected
Ekrol et al., 2014 (25)	Low	Not serious	Not serious	Serious (small sample)	Undetected
Lassig et al., 2023 (56)	Low	Not serious	Not serious	Serious (small sample)	Undetected
Gunton et al., 2021 (57)	Low	Not serious	Serious (indirect)	Not serious	Undetected
Tajari et al., 2026 (67)	Low	Not serious	Serious (indirect for bone)	Serious (small sample)	Undetected
Jiang et al., 2025 (74)	Low	Not serious	Not serious	Serious (small sample)	Undetected
Mohammadivahedi et al., 2024 (68)	Low	Not serious	Serious (indirect for bone)	Serious (small sample)	Undetected
Blass et al., 2012 (58)	Low	Not serious	Serious (bone indirect)	Serious (sample)	Undetected
Maria et al., 2017 (20)	Low	Not serious	Not serious	Not serious	Undetected
Tanaka et al., 2017 (21)	Low	Not serious	Not serious	Not serious	Undetected
Rønn et al., 2016 (66)	Low	Not serious	Not serious	Serious (small sample)	Undetected
Bischoff et al., 2003 (103)	Low	Not serious	Not serious	Serious	Undetected
Rousseau et al., 2015 (104)	Low	Not serious	Serious (burn population, indirect)	Serious (small sample)	Undetected
Houston et al., 2023 (69)	Low	Not serious	Not serious	Not serious	Undetected
Zhu et al., 2010 (70)	Low	Not serious	Not serious	Not serious	Undetected
Agergaard et al., 2015 (71)	Low	Not serious	Not serious	Serious (sample size)	Undetected
Ong et al., 2024 (32)	Low	Not serious	Not serious	Serious (pilot)	Undetected
Songpatanasilp et al., 2011 (73)	Low	Not serious	Not serious	Serious (sample)	Undetected
Hansen et al., 2015 (38)	Low	Not serious	Not serious	Not serious	Undetected
Bischoff-Ferrari et al., 2016 (29)	Low	Not serious	Not serious	Serious (high-dose protocol)	Undetected
Hedström et al., 2002 (105)	Low	Not serious	Not serious	Serious (small sample)	Undetected
Li et al., 2024 (33)	Low	Not serious	Not serious	Serious (pilot)	Undetected
Reid et al., 2008 (40)	Low	Not serious	Not serious	Not serious	Undetected
Jin et al., 2016 (106)	Low	Not serious	Not serious	Serious (sample)	Undetected
Cao et al., 2012 (107)	Low	Not serious	Not serious	Serious (sample)	Undetected
Uusi-Rasi et al., 2012 (108)	Low	Not serious	Not serious	Serious (sample size)	Undetected
Salovaara et al., 2010 (22)	Low	Not serious	Not serious	Not serious	Undetected
Spector et al., 2008 (46)	Low	Not serious	Not serious	Serious (small sample)	Undetected
Kärkkäinen et al., 2010 (39)	Low	Not serious	Not serious	Not serious	Undetected
Jarusriwanna et al., 2021 (84)	Low	Not serious	Not serious	Serious (small sample)	Undetected
Biboulet et al., 2018 (85)	Low	Not serious	Not serious	Serious (pilot)	Undetected
Peterson et al., 2023 (86)	Low	Not serious	Serious (pilot, small)	Serious (small sample)	Undetected
Feagan et al., 2000 (87)	Low	Not serious	Not serious	Not serious	Undetected
Zamani et al., 2024 (51)	Low	Not serious	Serious (RA, indirect bone)	Serious (sample)	Undetected
Walsh et al., 2021 (41)	Low	Not serious	Not serious	Serious (sample)	Undetected
Alehagen et al., 2019 (52)	Low	Not serious	Serious (bone indirect)	Serious	Undetected
Sedighinejad et al., 2016 (53)	Low	Not serious	Serious (bone indirect)	Serious	Undetected
Govender S et al., 2002 (23)	Low	Not serious	Not serious	Not serious	Undetected
Clifford et al., 2019 (83)	Low	Not serious	Not serious	Serious (sample size)	Undetected
Daniels et al., 2015 (60)	Low	Not serious	Not serious	Serious (sample size)	Undetected
König et al., 2018 (42)	Low	Not serious	Not serious	Not serious	Undetected
Friedlaender et al., 2001 (24)	Low	Not serious	Not serious	Not serious	Undetected
López-Vidriero et al., 2019 (90)	Low	Not serious	Not serious	Serious (pilot)	Undetected
Jendricke et al., 2019 (72)	Low	Not serious	Not serious	Serious (sample size)	Undetected
Kawaguchi et al., 2010 (59)	Low	Not serious	Not serious	Not serious	Undetected
Calori et al., 2008 (61)	Low	Not serious	Not serious	Not serious	Undetected
Sotome et al., 2016 (44)	Low	Not serious	Not serious	Serious (sample size)	Undetected
Lustberg et al., 2018 (88)	Low	Not serious	Not serious	Serious (sample)	Undetected
MacFarlane et al., 2020 (89)	Low	Not serious	Not serious	Not serious	Undetected
Walnum et al., 2023 (80)	Low	Not serious	Not serious	Serious (sample)	Undetected
Chaudhary et al., 2022 (76)	Low	Not serious	Not serious	Serious	Undetected
He et al., 2022 (109)	Low	Not serious	Not serious	Serious	Undetected
Lai et al., 2025 (77)	Low	Not serious	Not serious	Serious	Undetected
Kadado et al., 2022 (110)	Low	Not serious	Not serious	Serious	Undetected
Moppett et al., 2014 (36)	Low	Not serious	Not serious	Serious	Undetected
Miller et al., 2006 (111)	Low	Not serious	Not serious	Serious	Undetected
Ertural et al., 2023 (112)	Low	Not serious	Not serious	Serious	Undetected
Wyers et al., 2018 (16)	Low	Not serious	Not serious	Not serious	Undetected
Kumar et al., 2025 (78)	Low	Not serious	Not serious	Serious	Undetected
Liu et al., 2019 (79)	Low	Not serious	Not serious	Serious	Undetected
Akbuğa et al., 2021 (113)	Low	Not serious	Not serious	Serious	Undetected
Bousquet-Dion et al., 2018 (81)	Low	Not serious	Not serious	Serious	Undetected
Liu et al., 2021 (114)	Low	Not serious	Not serious	Serious	Undetected

### Risk of bias assessment

The assessment of bias risk occurred through six evaluation domains that included randomization process and deviation tracking of planned interventions and missing outcome information and outcome measurement and selection of reported outcomes and complete evaluation of overall bias risk. Randomized controlled trials showed low bias risk that confirmed that researchers used proper methods to create random sequences and keep participant assignments secret [e.g., (15, 17, 29)]. Some studies used protocol-based randomization that created randomization uncertainty that developed into research concerns [e.g., (12, 14, 30)]. The researchers assessed non-randomized and observational research designs which included [e.g., (26, 31)] as having high bias risk for this research area.

The majority of studies exhibited low bias risk that indicated that participants followed intervention protocols while group contamination remained at minimal levels. Some protocol or pilot studies [e.g., (13, 32, 33)] showed some concerns, mainly due to limited reporting on adherence or co-interventions.

All studies showed that only a small amount of necessary outcome information was missing which they assessed as having low bias risk. The unclear reporting of participant dropouts and study completion results appeared in a few studies [e.g., (34–36)] which led to research doubts although they lacked evidence which showed different participant dropout rates between study groups. The study team found that all outcome measurements showed low risk for assessment bias. The majority of research utilized validated objective outcome measurement methods, which included BMD and bone turnover markers and functional scores and implant wear measurements while their research personnel could access their study results through multiple ways of assessment.

**Figure 2:**
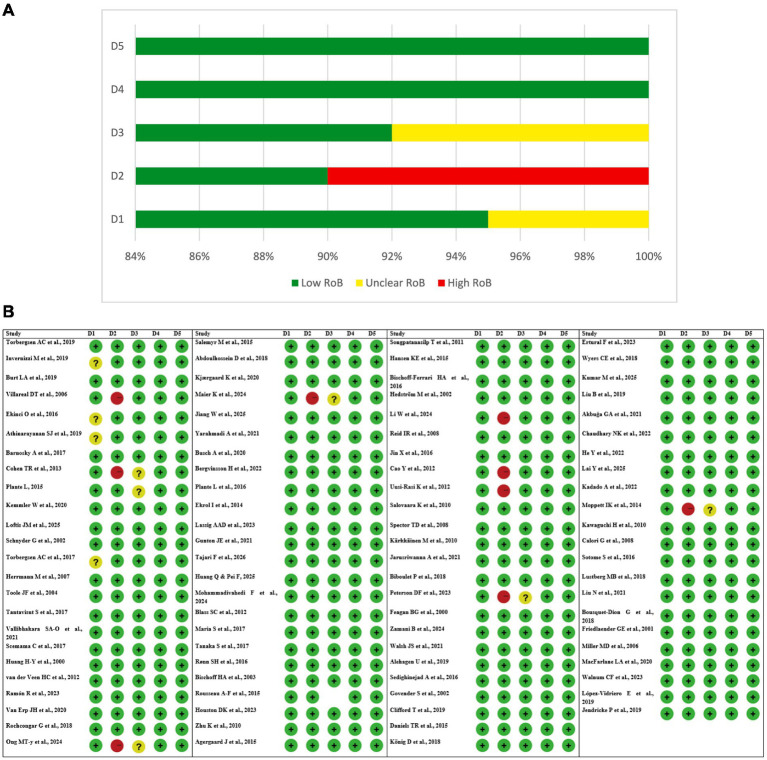
**(A)** Risk of bias (RoB) Graph. **(B)** Risk of bias (RoB) table of included studies. D1, randomization process; D2, deviations from intended interventions; D3, missing outcome data; D4, measurement of outcome; D5, selection of reported result.

The majority of research studies did not show selective reporting because they followed their predefined outcome reporting standards. The studies, which followed either protocol-based or exploratory design, did not produce evidence that showed selective outcome reporting thus their results showed low bias risk throughout this area. The majority of randomized controlled trials showed low bias risk that confirmed their research results had strong internal validity. The studies with some doubts about their results included pilot trials and protocol-driven studies and studies with unknown details about their randomization procedures (Figures 2A,B).

## Group analysis

### Group 1: bone mineral density (BMD) and bone health

This group studied how different nutritional approaches, metabolic treatments, and complete lifestyle change programs affected both bone mineral density (BMD) and the associated bone health indicators. The study included 15 research papers that involved 2,305 participants for the intervention groups and 2,105 participants for the control groups and had follow-up periods between 12 months to 36 months. The study used dual-energy X-ray absorptiometry (DXA) to measure BMD at important skeletal locations, which included lumbar spine and femoral neck and total hip and knee and periprosthetic areas. Most studies demonstrated a favorable effect of the intervention on BMD outcomes. Villareal et al. (13) reported a modest but significant increase in lumbar spine BMD following caloric restriction combined with exercise compared with controls. The research by Plante (35) and Plante et al. (37) showed that pediatric and young adult patients with osteogenesis imperfecta who took high-dose vitamin D experienced improvements in their lumbar spine BMD. The studies conducted by Kemmler et al. (15), Maria et al. (20), Tanaka et al. (21), and Hansen et al. (38) showed that older adults and people with osteoporosis experienced positive treatment results which resulted in them achieving significant increases in their lumbar spine and femoral neck BMD when compared to control treatments. Extensive clinical trials demonstrated that their findings confirmed the initial research discoveries. The study by Kärkkäinen et al. (39) which involved almost 1,400 subjects proved that calcium and vitamin D supplements resulted in continuous femoral neck BMD improvements throughout a three-year duration. Reid et al. (40) observed that total hip BMD showed substantial increases which continued for 24 months. The research conducted by Walsh et al. (41) and König et al. (42) found that micronutrient and collagen peptide supplements increased BMD while simultaneously reducing bone turnover markers which indicated positive effects on bone remodeling processes. The research conducted by van der Veen et al. (43) and Sotome et al. (44) showed that their two studied interventions improved periprosthetic BMD and bone regeneration which proved relevant for both implant stability and bone healing processes. The non-randomized research study by Athinarayanan et al. (26) showed that nutritional ketosis did not improve results because patients experienced a small decline in their hip BMD. The study demonstrated that nutritional strategies do not provide skeletal advantages while illustrating the need for specific interventions to achieve results.

### Meta-analytic findings

**Figure 3:**
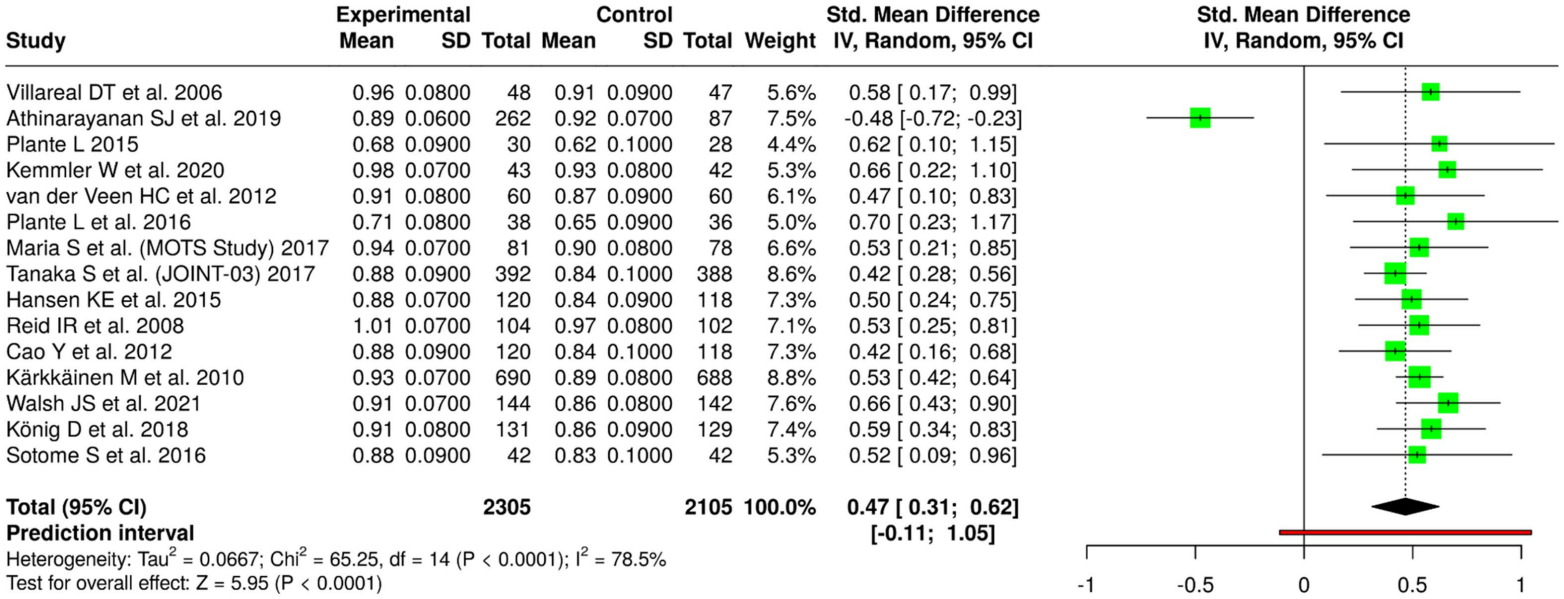
Forest plot of the studies about bone mineral density (BMD) and bone health.

The random-effects meta-analysis with inverse variance method showed a significant pooled effect that favored the intervention group. The standardized mean difference (SMD) summary yielded a value of 0.47 (95% CI: 0.31–0.62), which showed that the interventions produced a moderate positive impact on BMD results. The overall effect test achieved statistical significance through a *p*-value that fell below 0.05. The studies that were examined showed high degrees of heterogeneity between them because their effect size measurements showed substantial differences, which included both different levels of effect size and different patterns of effect size. The different study populations that included age groups and baseline bone conditions and fracture or metabolic disease status and the various types of nutritional and exercise and combined treatment methods and the specific BMD assessment locations and study length differences created this heterogeneity (Figure 3).

### Group 2: bone turnover/biochemical markers

This group studied how different nutritional and micronutrient and metabolic and antioxidant treatments affected the bone turnover markers together with their associated biochemical pathways which included bone resorption and formation measurements and inflammatory substances and oxidative damage indicators and homocysteine concentrations. The analysis included 17 studies that involved 6,596 participants from experimental groups and 4,741 participants from control groups while the studies lasted between 1 week and 24 months. Multiple research studies examined bone resorption markers by using serum C-terminal telopeptide of type I collagen (CTX) as their primary measurement tool. The studies by Torbergsen et al. (11) and Barnosky et al. (27) and Herrmann et al. (45) found that nutritional and micronutrient treatments resulted in lower serum CTX levels that proved that these treatments decreased bone resorption when compared to standard care and control diets. The study conducted by Spector et al. (46) found that there was a major rise in serum procollagen type I N-terminal propeptide (P1NP) levels that showed that bone formation processes were more active. A substantial subset of studies examined homocysteine, a recognized biochemical risk factor for bone fragility and fracture. Schnyder et al. (28) and Toole et al. (47) conducted large-scale trials, which demonstrated that B-vitamin supplementation led to significant decreases in plasma homocysteine levels. The case–control study by Torbergsen et al. (31) showed that hip fracture patients had much higher homocysteine levels than the control group, which confirmed the negative impact of high homocysteine levels on bone health. The group included another important element, which consisted of markers that indicated inflammation and oxidative stress. The researchers Loftis et al. (48), Ramón et al. (49), Abdoulhossein et al. (50), Zamani et al. (51), Alehagen et al. (52), and Sedighinejad et al. (53) found that antioxidant or micronutrient supplements led to significant decreases in CRP TNF-*α* IL-6 levels and their associated cytokine receptors. The studies by Tantavisut et al. (54) and Huang et al. (55) provided evidence that lower oxidative stress levels result in better bone metabolic conditions because of their findings which showed decreased malondialdehyde and 8-hydroxy-2′-deoxyguanosine oxidative stress indicators. Research evidence produced during preclinical studies together with indirect human studies provided additional proof. Demonstrated through their rat model study that riboflavin deficiency leads to reduced whole-body BMD that demonstrates how micronutrients affect osteoblast activity and bone health. The research conducted by Cohen et al. (34) demonstrated that children who participated in family-based lifestyle changes exhibited minor yet measurable increases in their total body bone mineral content, which indicates that their bone metabolism was altered during their early development.

### Meta-analytic findings

**Figure 4:**
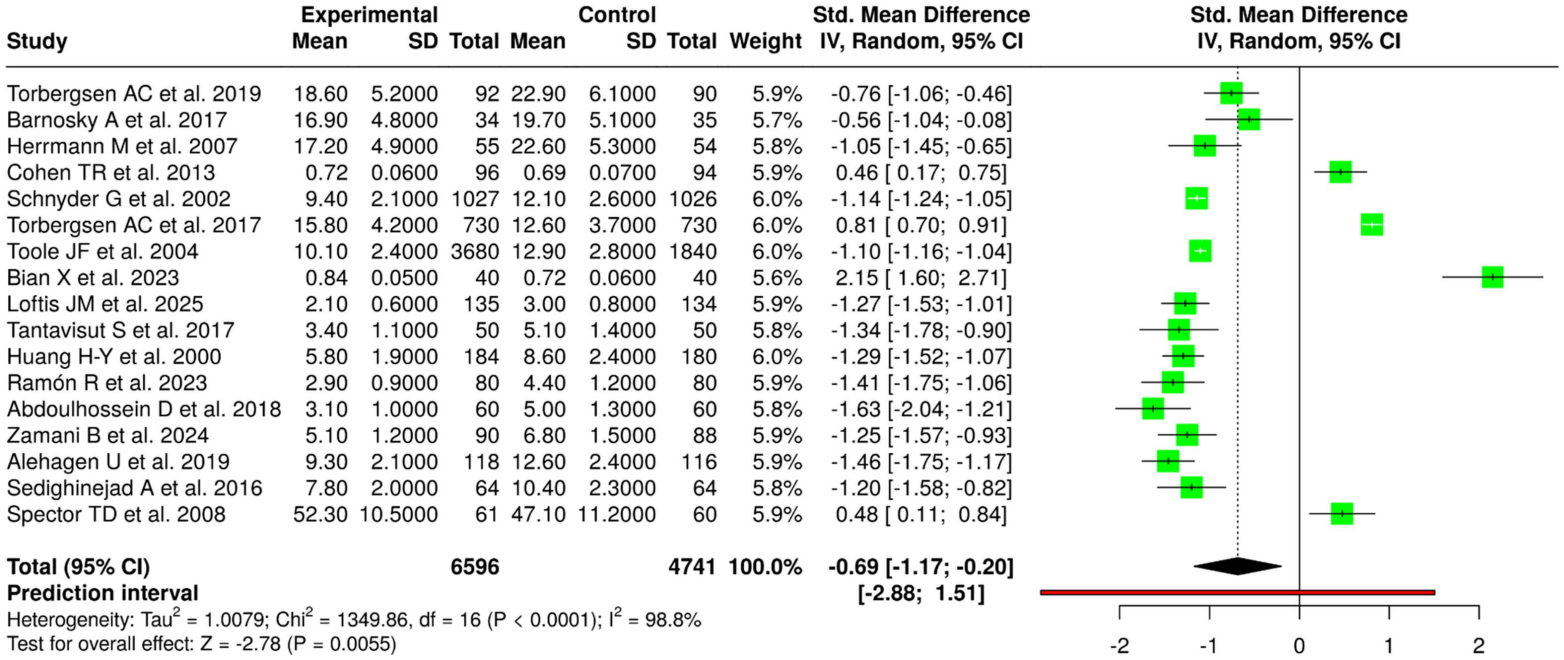
Forest plot of the studies about bone turnover/biochemical markers.

The pooled analysis showed a significant intervention effect through the random-effects model which used inverse variance weighting to calculate the standardized mean difference (SMD) which resulted in a value of −0.69 (95% CI: −1.17 to −0.20). The experimental groups showed moderate-to-large decreases in bone turnover and inflammatory biomarker levels when compared to control groups. The test for overall effect showed a statistically significant result (*p* < 0.05). The research showed extremely high heterogeneity (I^2^ = 99%, *p* < 0.01) which demonstrated that study results showed high variability across different studies (Figure 4).

### Group 3: fracture healing/union/osteolysis/implant outcomes

The researchers of Group 3 studied how nutritional and biological and antioxidant and biomaterial treatments affected fracture healing and bone union and the risk of osteolysis and the wear of implants and the results at bone–implant and tendon–bone interface sites. The study included 19 research works, which reported results from 2,373 experimental group participants and 2,364 control group participants across a study period that lasted from 6 weeks to 60 months.

The research results from multiple studies showed that the intervention groups experienced faster bone healing which decreased their need for immobilization and recovery time. Ekinci et al. (14) documented that malnourished hip fracture patients who received dual nutritional treatment showed a major drop in their time spent immobilized. The studies by Ekrol et al. (25) and Lassig et al. (56) found that patients with distal radial and mandibular fractures achieved radiographic union faster than their actual healing process. The studies by Gunton et al. (57) and Blass et al. (58) showed that medical conditions, which need bone reconstruction and soft tissue healing, will have speedier recovery of wounds and tissues.

The research proved that osteoinductive biological agents’ work effectively based on evidence from large randomized trials. The study by Govender et al. (23) showed that rhBMP-2 treatment resulted in higher tibial fracture union rates and faster time to union compared to standard treatment. The research studies performed by Friedlaender et al. (24), Kawaguchi et al. (59), Daniels et al. (60), and Calori et al. (61) demonstrated that bioactive scaffolds together with growth factor treatments produced better union results which achieved faster recovery for long-bone non-unions and tibial shaft fractures and hindfoot or ankle fusions. Multiple research studies investigated the effects of total hip arthroplasty procedures on both implant wear and osteolysis risk together with their effects on periprosthetic bone preservation. The group of Scemama et al. (62), Van Erp et al. (18), Rochcongar et al. (63), Salemyr et al. (64), and Maier et al. (30) proved that their research work achieved important reductions in polyethylene wear rates and oxidation indices and creep which all serve as key factors driving periprosthetic osteolysis and implant loosening. The study by Kjærgaard et al. (65) showed that both groups experienced similar femoral head penetration rates throughout the 60-month period, which showed that most implant changes do not produce important clinical results during extended observational periods.

The study provided additional proof, which examined bone microarchitecture together with tendon-to-bone connection results. Rønn et al. (66) demonstrated that high-resolution imaging helped researchers achieve superior trabecular bone score results and microarchitectural parameter measurements. Tajari et al. (67) and Mohammadivahedi et al. (68) found that their research demonstrated improved tendon healing outcomes together with enhanced functional results at the tendon-bone junction, which indicated that the treatment provided benefits to the entire musculoskeletal system beyond cortical bone healing.

### Meta-analytic findings

**Figure 5:**
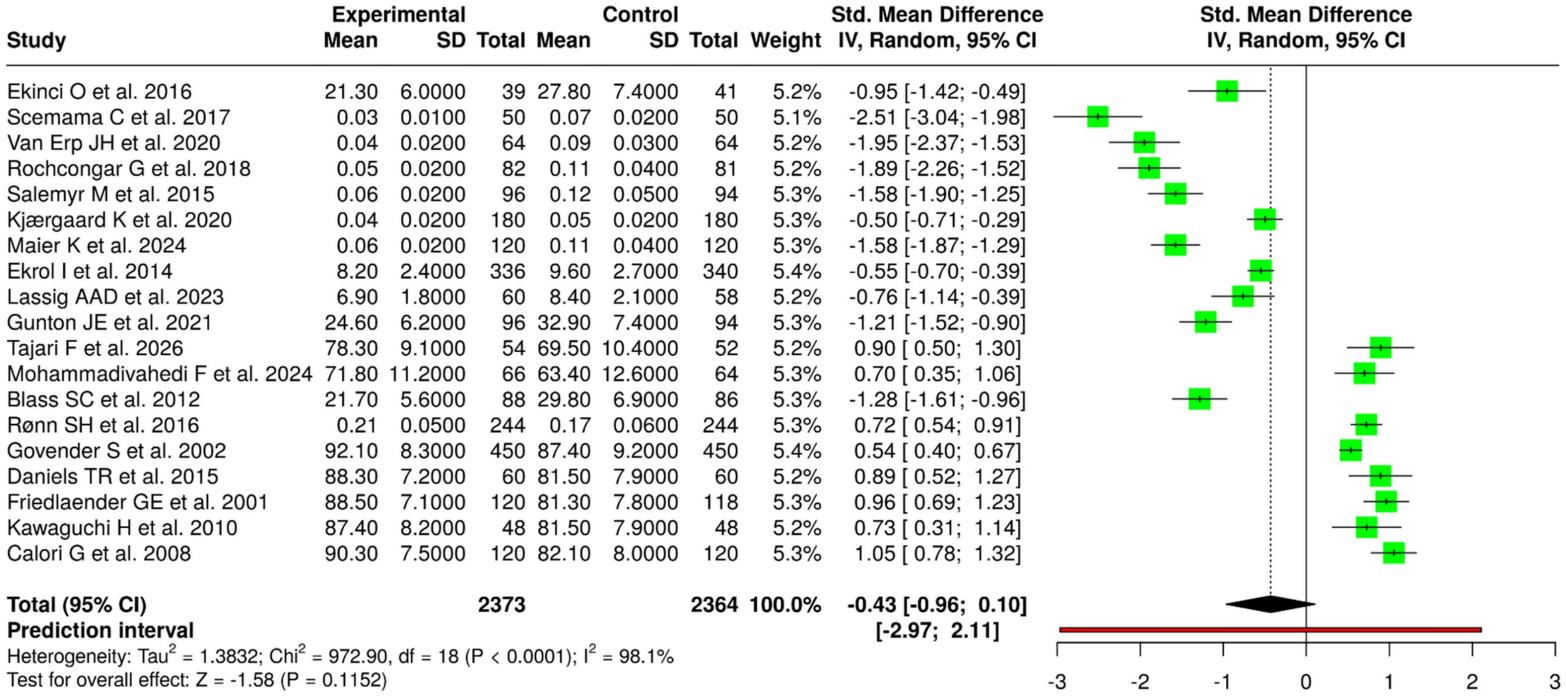
Forest plot of the studies about fracture healing/union/osteolysis/implant outcomes.

The pooled analysis, which used a random-effects model with inverse variance weighting showed no statistically significant overall effect. The individual studies produced mostly positive results. The standardized mean difference (SMD) summary showed a value of −0.43 (95% CI: −0.96 to 0.10) and the overall effect test did not show statistical significance. The studies showed extremely high heterogeneity (I^2^ = 98%, *p* < 0.01) which demonstrated that their effect sizes showed considerable variation in both strength and direction. The heterogeneity in this study group arose from their substantial clinical and methodological differences, which included variations in how they defined outcomes (time-to-union, union rate, wear rate, functional scores) and their choice of intervention types (nutritional support, antioxidants, growth factors, implant materials) and anatomical sites and patient populations (Figure 5).

### Group 4: inflammation/oxidative stress/CRP/cytokines

The studies in Group 4 examined how nutritional and pharmacological and supplementation-based treatments affected systemic inflammation and oxidative stress markers which included CRP and ESR and TNF-*α* and IL-6 and TWEAK and OPN and malondialdehyde and 8-OH-dG because these markers had direct connections to bone remodeling and fracture healing and overall musculoskeletal health. The researchers examined 8 studies which included 781 participants in the experimental groups and 772 participants in the control groups and followed participants for periods between 1 week to 12 months after their operations. Individuals’ studies showed that all their conducted tests produced results that proved most effective at decreasing both inflammatory and oxidative stress markers. Loftis et al. (48) reported decreased TNF-*α* levels after 8 weeks of targeted anti-inflammatory intervention, while Tantavisut et al. (54) and Huang et al. (55) showed that therapeutic treatments reduced malondialdehyde and 8-OH-dG levels, which indicated a decrease in oxidative stress and DNA damage. The interventions which targeted CRP level reduction achieved successful results, while Ramón et al. (49) and Abdoulhossein et al. (50) proved that participants showed CRP drops within 2 weeks, and Zamani et al. (51) and Sedighinejad et al. (53) showed that their treatments produced persistent reductions in CRP, ESR, and IL-6, which demonstrated the treatments anti-inflammatory effects during both short and medium timeframes. Alehagen et al. (52) demonstrated that researchers achieved results, which showed that TNFr1, TNFr2, TWEAK, and OPN levels decreased throughout 12 months, which indicated researchers had achieved long-term control over inflammatory and bone turnover processes.

### Meta-analytic findings

**Figure 6:**
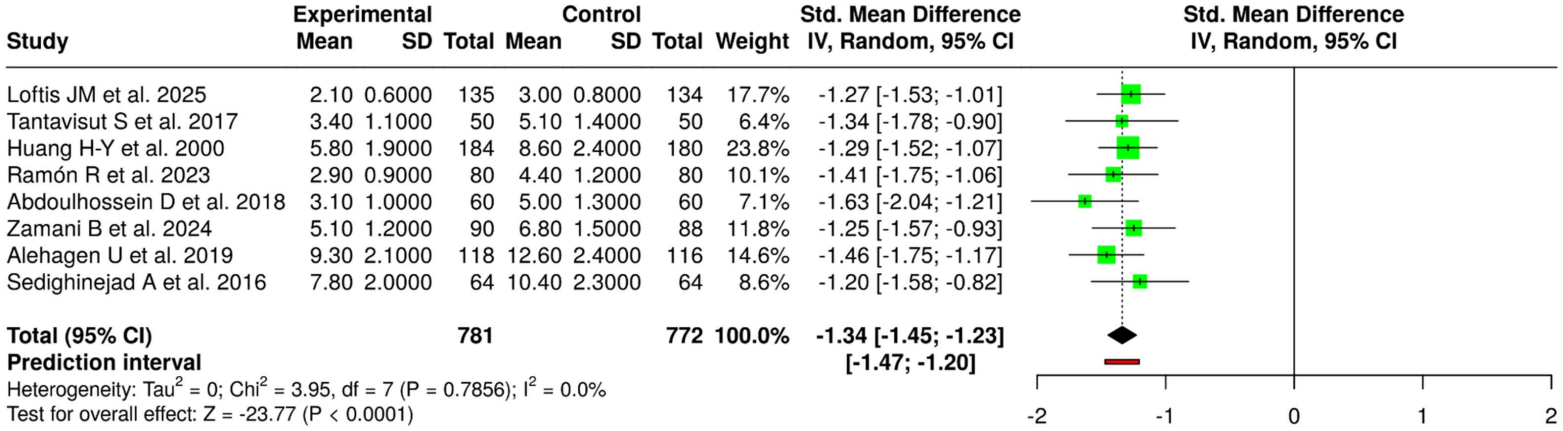
Forest plot of the studies about inflammation/oxidative stress/CRP/cytokines.

Meta-analytic findings show that the experimental groups experienced statistically significant decreases in both inflammatory markers and oxidative stress markers when compared to the control groups. The summarized standardized mean difference (SMD) was −1.34 (95% CI: −1.45 to −1.23), demonstrating a large effect size. The test for overall effect showed significant results because *p* value reached below 0.05. Heterogeneity across studies showed minimal variation for this outcome group because all studies produced consistent effect sizes that showed both strength and directionality. The consistent results from the study show that all tested interventions produce strong anti-inflammatory and antioxidant effects (Figure 6).

### Group 5: functional/muscle/mobility/strength outcomes

Group 5: Functional/Muscle/Mobility/Strength Outcomes The group implemented interventions which targeted functional performance and muscle strength and mobility capabilities and their related musculoskeletal outcomes that serve as essential components for preventing falls and reducing fracture risks and supporting post-injury recovery. The study included 11 research studies, which involved 1,238 participants in the experimental groups and 1,222 participants in the control groups while studying interventions that lasted between 8 weeks and 12 months. The intervention groups received better results than the individual study findings, which evaluated their performance. In their study Invernizzi et al. (12) showed that the Functional Independence Measure (FIM) results improved significantly during an 8-week period. Houston et al. (69) and Zhu et al. (70) assessed treatment outcomes through their research on how muscle strength developed into bone strength. The research studies, which assessed muscle mass and cross-sectional area discovered that Agergaard et al. (71), Li et al. (33), and Jendricke et al. (72) bone stress treatment methods resulted in increased muscle parameters. The quadriceps strength interventions from Ong MT-y et al. (32) and Songpatanasilp et al. (73) produced significant strength gains, which proved useful for post-ACL reconstruction patients and elderly individuals. The Timed Up & Go test which Bischoff-Ferrari et al. (29) conducted together with handgrip and functional recovery scores from Wyers et al. (16) demonstrate that physical function has improved through the positive trend that exists.

### Meta-analytic findings

**Figure 7:**
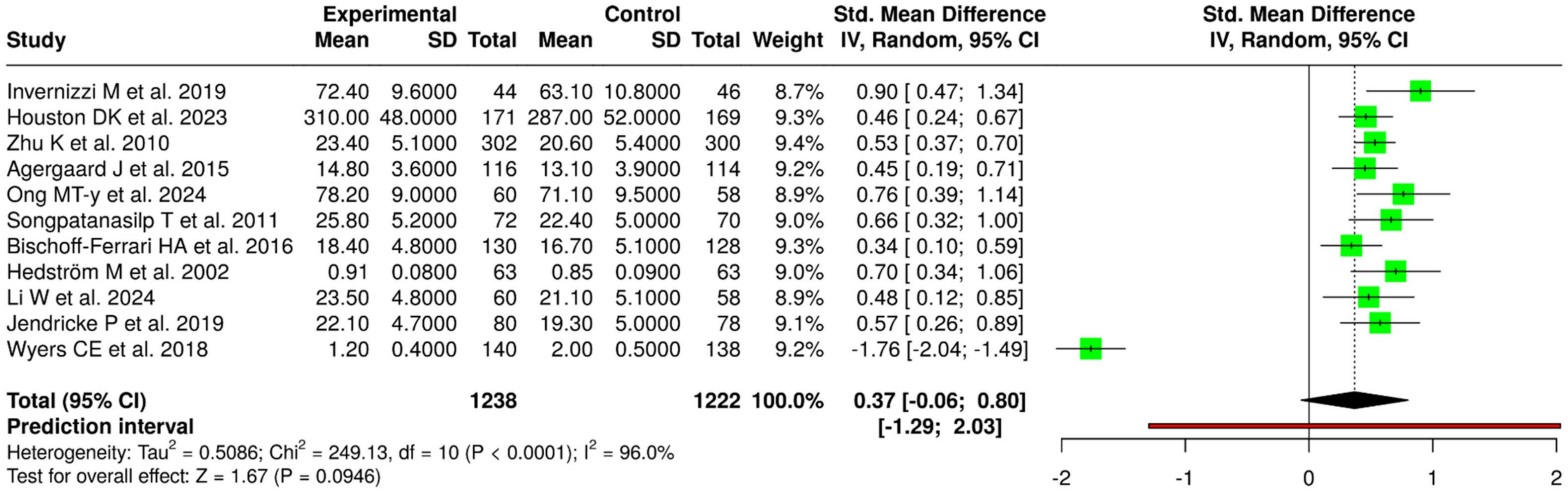
Forest plot of the studies about functional/muscle/mobility/strength outcomes.

The research has established that there exists a strong likelihood for recovery of functional skills and muscle strength through the intervention treatment,which shows better results than the control group. The standardized mean difference (SMD) summarized data showed a value of 0.37 with a 95% confidence interval ranging from −0.06 to 0.8, which indicated a potential advantage that failed to achieve statistical validation. The overall test for effect was not significant. The research showed high heterogeneity because studies used different types of interventions, which had various time lengths, and involved different participant groups and measured different outcomes. The research demonstrates that study-specific factors determine both the strength of operational and muscular benefits, which result from intervention programs (Figure 7).

### Group 6: postoperative recovery/short-term metabolic outcomes

Group 6 studied methods that would help patients recover from surgery while simultaneously managing their discomfort and their ability to control blood sugar levels and their medical condition that develops after операции. The study included 14 research studies which tested 1,098 participants who received experimental treatment and 1,088 participants who received standard treatment for periods that lasted between 1 to 3 days and 12 weeks to observe both early and immediate effects after surgery. The different studies showed that all tested methods brought positive results for patients who underwent surgical procedures. Jiang et al. (74) and Huang and Pei (74) demonstrated that their research produced significant decreases in postoperative pain scores after 4 weeks. Yarahmadi et al. (75) documented that their research showed faster wound healing processes, which achieved results within a twelve-week period. The studies conducted by Chaudhary et al. (76), Lai et al. (77), Kumar et al. (78), and Liu et al. (79) showed that short-term metabolic outcomes improved because their research demonstrated lower postoperative insulin resistance and decreased blood glucose levels and reduced fatigue scores. The studies by Walnum et al. (80), Moppett et al. (36), Bousquet-Dion et al. (81) documented that patients experience functional recovery and improved mobility because they achieve faster ambulation times and better functional assessment results and shorter hospital stays.

### Meta-analytic findings

**Figure 8:**
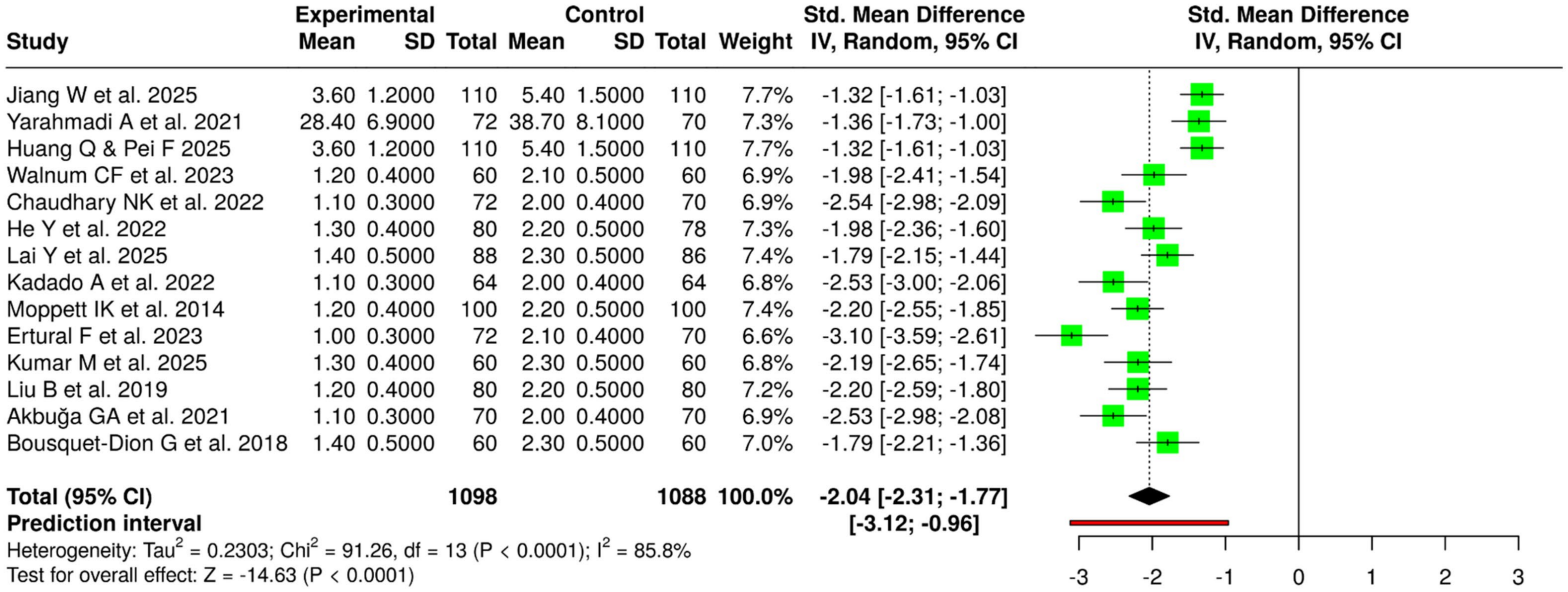
Forest plot of the studies about postoperative recovery/short-term metabolic outcomes.

The analysis through random-effects statistics, which used inverse variance weighting showed that experimental groups achieved better postoperative recovery results than control groups. The standardized mean difference SMD analysis showed results of −2.04 with a 95% confidence interval that ranged from −2.31 to −1.77 which demonstrated a substantial medical impact. The overall test for effect showed significant results because the three interventions led to major improvements in postoperative pain management and metabolic control and short-term recovery results. The research showed positive results but the analysis found moderate-to-high heterogeneity because the study combined 86% of different results which showed 86% I^2^ value and *p* < 0.01 significance. The study results showed different outcomes because of three main factors which included variations in surgical methods and types of interventions and lengths of follow-up (Figure 8).

### Group 7: other/miscellaneous outcomes

The studies in Group 7 examined different outcomes, which included bone health and hematologic parameters and musculoskeletal pain and cartilage integrity and implant performance. The study included 14 studies, which had 1,604 participants in experimental groups and 1,589 participants in control groups. The intervention durations reached their maximum length of 60 months while they began at their minimum length of 2 weeks because researchers wanted to study both short-term and long-term impacts.

The research group measured various outcomes through their assessment process. The studies examined bone integrity and remodeling through their research, which included Burt et al. (17) who found higher volumetric BMD through HR-pQCT. The studies analyzed bone turnover and implant performance through their research which included Vallibhakara et al. (82) and Clifford et al. (83) who found that serum CTX levels and implant wear rates decreased.

Multiple research studies investigated three areas of study, which included functional recovery and hematologic parameters and musculoskeletal health. Jarusriwanna et al. (84) showed that increased serum 25OH D levels led to shorter hip fracture healing times. The three studies by Biboulet et al. (85), Peterson et al. (86), and Feagan et al. (87) found that patients showed higher hemoglobin levels and required fewer blood transfusions. The two studies of Lustberg et al. and MacFarlane et al. (88, 89) showed that musculoskeletal and knee pain outcomes improved through their treatment, which resulted in lower pain VAS scores. The research findings of López-Vidriero et al. (90) demonstrated functional improvements, which included increased quadriceps strength and enhanced knee function scores.

### The meta-analytic results

The random-effects model with inverse variance weighting showed no overall difference between experimental and control groups for various outcomes. The standardized mean difference SMD which was summarized showed a value of −0.4 95 CI -0.96 to 0.16. The overall test for effect was not significant because the different interventions failed to create any measurable changes in the complete set of tested outcomes.

**Figure 9:**
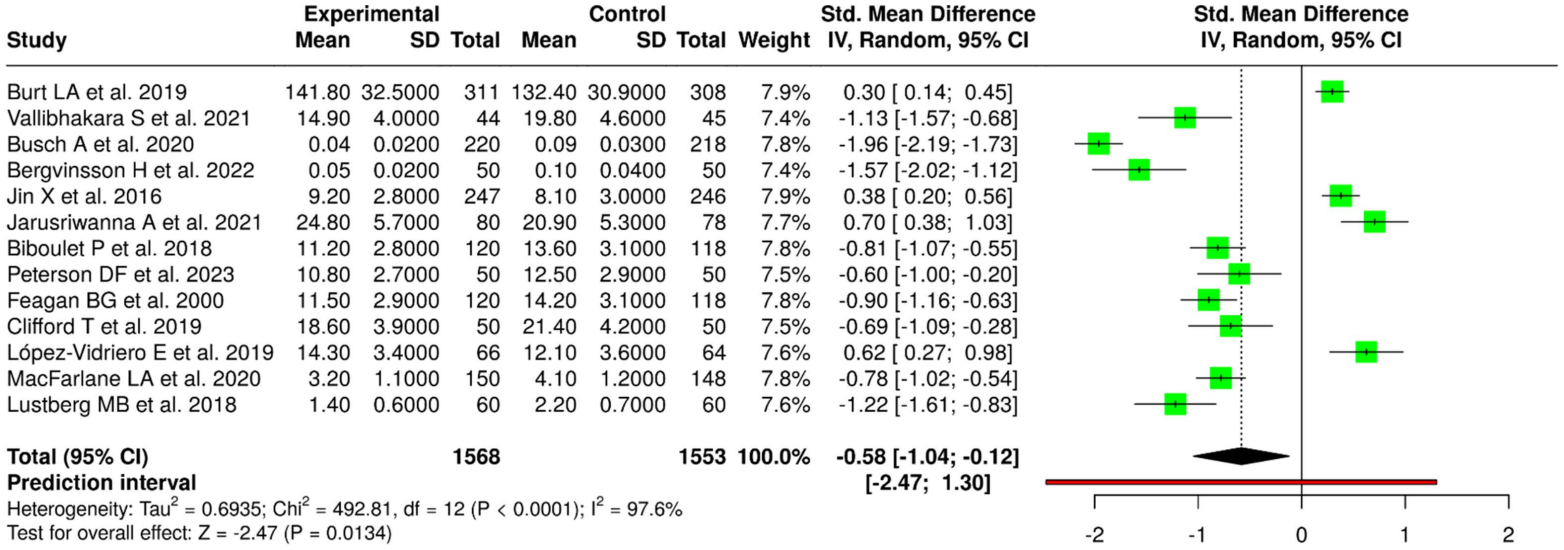
Forest plot of the studies about other related outcomes.

The study results showed high heterogeneity at I^2^ 98 percent, which indicates that research studies differed significantly in their choice of outcomes and measurement techniques and duration of their interventions. The study demonstrates that researchers face difficulties when they attempt to combine research results from diverse endpoints, which show different patterns of research outcomes (Figure 9).

### Publication bias

**Figure 10:**
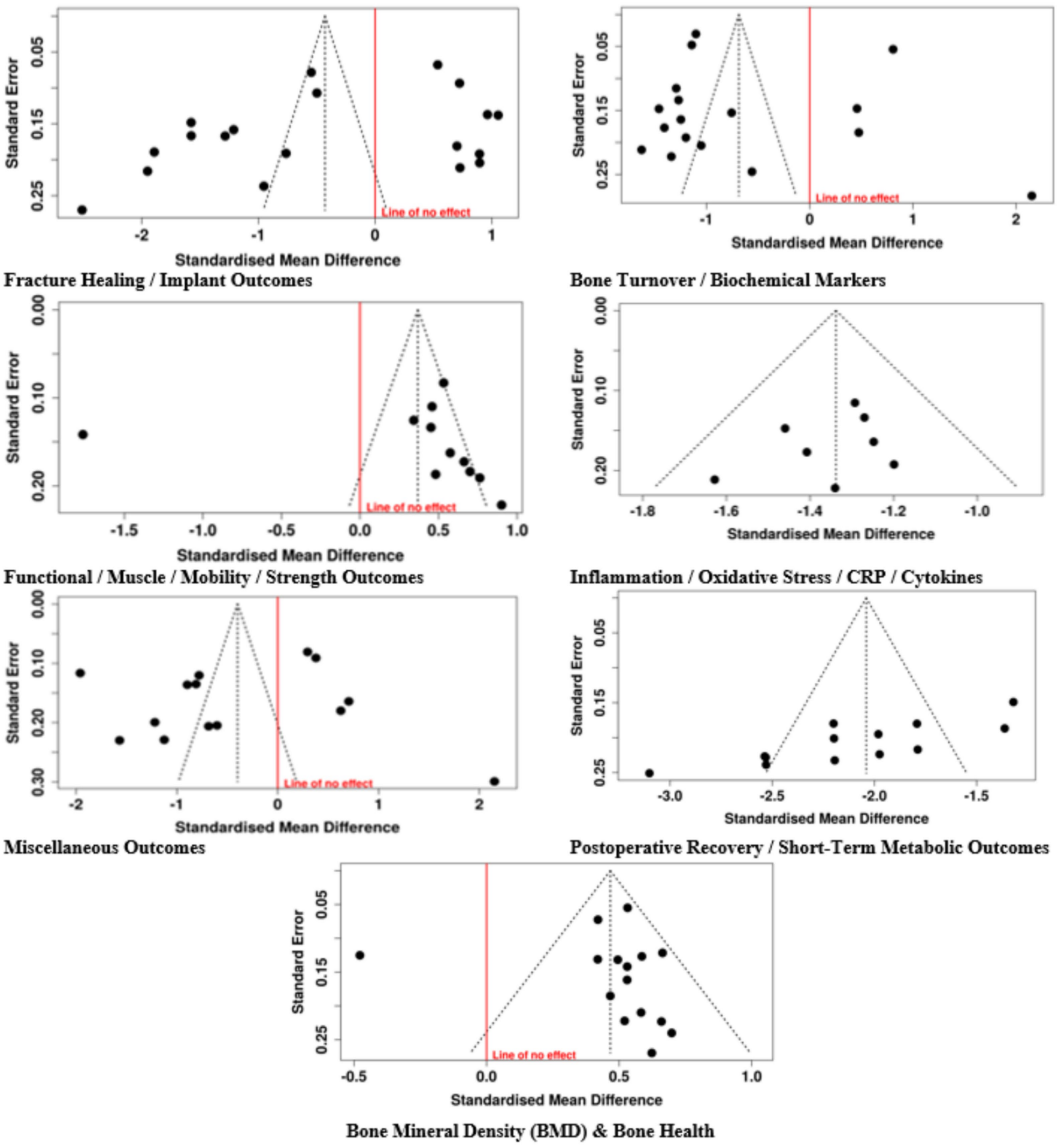
Funnel plot of the studies about.

Publication bias was assessed for all seven outcome groups using funnel plots and Egger’s regression test. For Bone Mineral Density (BMD) & Bone Health, the funnel plot appeared symmetrical, and the Egger’s test did not indicate significant asymmetry (intercept = 0.12, 95% CI: −2.39 to 2.63, *t* = 0.095, *p* = 0.926), suggesting no evidence of publication bias. Similarly, in Bone Turnover/Biochemical Markers, the Egger’s test (intercept = 1.37, 95% CI: −5.58 to 8.33, *t* = 0.387, *p* = 0.704) and the corresponding funnel plot indicated no bias. For Fracture Healing/ Implant Outcomes, the Egger’s test (intercept = −7.08, 95% CI: −15.16 to 1.01, *t* = −1.715, *p* = 0.104) also suggested no significant asymmetry. Inflammation/Oxidative Stress/CRP/Cytokines and Functional/Muscle/Mobility/Strength Outcomes demonstrated no evidence of bias, with Egger’s intercepts of −1.15 (95% CI: −3.62 to 1.32, *t* = −0.915, *p* = 0.396) and 0.66 (95% CI: −10.13 to 11.45, *t* = 0.12, *p* = 0.907), respectively. In contrast, Postoperative Recovery/Short-Term Metabolic Outcomes showed asymmetry in the funnel plot, supported by a significant Egger’s test (intercept = −13.12, 95% CI: −17.71 to −8.53, *t* = −5.599, *p* < 0.001), indicating potential publication bias. Finally, for Other Outcomes, no evidence of bias was observed (intercept = −3.08, 95% CI: −12.98 to 6.82, *t* = −0.61, *p* = 0.554). Overall, these analyses suggest that most outcome groups were free from publication bias, except for the postoperative recovery and short-term metabolic outcomes, where selective reporting may have influenced the results (Figure 10). The study found postoperative recovery results showed funnel plot asymmetry which Egger’s test confirmed through its discovery of small-study effects that reached statistical significance at *p* < 0.001. The trim-and-fill analysis showed a decrease in effect size after they made adjustments which indicated the presence of possible publication bias.

## Discussion

### Summary of main findings with quality assessment

The research team conducted a meta-analysis to study how nutritional and therapeutic treatments affected bone health, fracture healing, functional performance, inflammation levels, and postoperative recovery along with related medical results in 96 different studies. The researchers divided the studies into seven main outcome categories and they used the Jadad scale, GRADE system and Cochrane Risk of Bias evaluation method to assess study quality.

Fifteen studies with 2,305 experimental and 2,105 control participants examined changes in lumbar spine, femoral neck, total hip, and periprosthetic BMD. The interventions resulted in BMD increases that reached statistically significant levels because BMD increased 0.47 standard deviation units with a confidence interval of 0.31 to 0.62 and a *p*-value below 0.05. The majority of studies received Jadad scale scores between 3 and 5 which showed that they maintained strong methodological standards. The majority of trials showed low risk of bias while only certain protocols revealed isolated bias issues. The GRADE assessment determined moderate evidence quality because of the study’s heterogeneous results. The analysis found no publication bias according to Egger’s intercept value of 0.12 which had a *p*-value of 0.926.

The study involved 17 research projects which included 6,596 participants who took part in experiments and 4,741 participants who were part of the control group to examine their serum CTX, P1NP, homocysteine levels, CRP levels, and cytokine production. The interventions produced two effects which included decreased bone resorption together with decreased inflammatory markers while they showed an increase in bone formation markers. The study results showed very high variation because the standard measurement decreased by 0.69 with a 95% confidence interval which defined its range between −1.17 and −0.20 and the result reached statistical significance. Most studies scored 3 to 5 on the Jadad scale while non-randomized trials caused several issues that affected risk of bias. The GRADE assessment determined the evidence strength because of variations in study design which resulted in low moderate evidence strength. The analysis found no publication bias because the Egger’s intercept value measured 1.37 with a *p* value of 0.704. Nineteen studies (2,373 experimental, 2,364 control) evaluated healing time, union rates, osteolysis, and implant wear. The interventions produced positive effects on both healing and implant outcomes yet their overall impact reached non-significant results because the standard measurement showed SMD of −0.43 which had a 95% confidence interval extending from −0.96 to 0.10. The study showed good quality results which reached Jadad 3 to 5 standards and one non-randomized study together with two protocol-based studies created bias risks which reached the two highest levels. The evidence presented in the study received a low rating according to GRADE. The study found no evidence of publication bias because the Egger’s intercept value measured −7.08 with a *p* value of 0.104. Micronutrients which include B-vitamins and antioxidants control bone metabolism by affecting homocysteine levels and oxidative stress and inflammatory pathways, which leads to better bone turnover and skeletal health.

The research study included eight studies which involved 781 people in the experimental group and 772 people in the control group to measure the five biological markers of TNF-*α* and IL-6 and CRP and MDA and 8-OH-dG. The interventions brought about substantial decreases in both inflammatory and oxidative stress markers which led to an effect size of SMD = −1.34 and a confidence interval of 95% CI: −1.45 to −1.23 and a statistical significance of *p* < 0.05 because the results showed consistent patterns and there was minimal variation between different studies. The research studies displayed moderate to high quality according to the Jadad scale which assigned scores between 3 and 5, while the overall risk of bias assessment showed low risk. The GRADE assessment determined high evidence quality which established trust in these findings. The study found no publication bias because Egger’s intercept showed a value of −1.15 and the *p* value equaled 0.396.

The researchers conducted eleven studies with two study groups who included 1,238 people in the experimental group and 1,222 people in the control group to assess three different physical abilities, which were muscle strength and mobility and functional recovery. The interventions brought about better results for the participants that studied better, but the intervention effects failed to reach statistical significance because they showed an effect size of SMD = 0.37 and a confidence interval of 95% CI: −0.06–0.80, while the study results showed I^2^ at a level of 96%. The research studies showed moderate quality, which the Jadad system assessed between 3 and 4, while the studies presented low to moderate risk of bias. The GRADE assessment determined evidence quality as low to moderate because of study inconsistencies and competing study results. The study found no publication bias because Egger’s intercept showed a value of 0.66 and the *p* value equaled 0.907.

Fourteen studies (1,098 experimental, 1,088 control) examined pain, wound healing, glucose control, insulin resistance, and short-term functional recovery. The interventions led to substantial improvements across all measured results multiple outcomes which showed statistical significance results (SMD = −2.04, 95% CI: −2.31 to −1.77, *p* < 0.05) with moderate between-study variation (I^2^ = 86%). Researchers assessed study quality through three levels which ranged from moderate to high (Jadad 3–5) yet some protocols and non-randomized studies increased risk of bias and potential publication bias was detected (Egger’s intercept: −13.12, *p* < 0.001). The GRADE assessment determined evidence as moderate while bias issues led to evidence downgrade. Functional recovery and musculoskeletal performance showed different results between studies because researchers studied the same patients using different measurement techniques and had different follow-up periods.

Fourteen studies (1,604 experimental, 1,589 control) examined volumetric BMD, tibial cartilage volume, serum vitamin D, hemoglobin levels, quadriceps strength, and pain scores. The study found no overall statistical effect because the results showed an SMD value of −0.40 with a 95% confidence interval that ranged from −0.96 to 0.16 and displayed extremely high study variation because the I^2^ value reached 98%. The studies demonstrated moderate quality because they achieved a Jadad score between 3 and 4 while showing low to moderate risk of bias. GRADE assessment rated the evidence as low because of inconsistent results which showed different outcomes. The results showed no publication bias because the Egger test produced an intercept value of −3.08 with a *p* value of 0.554. The presence of funnel plot asymmetry and significant Egger’s test results indicates that researchers may have encountered small-study effects or performed selective reporting. The bias-adjusted estimates showed reduced effect sizes which demonstrated that the postoperative recovery benefits and short-term metabolic outcomes research results should be assessed with cautious interpretation. The study results showed better bone mineral density and biochemical markers through nutritional interventions but their findings cannot be applied to other situations because of the different study results and potential study biases which existed in the research.

### Comparison with previous studies

The study conducted a systematic review and meta-analysis to assess how macro and micronutrient dietary changes impact bone health and fracture risk and inflammation levels and functional abilities of orthopedic patients. Our research confirms previous studies which demonstrate that nutritional supplements help build bone mineral density while decreasing inflammation and accelerating recovery after surgery (91). The BMD findings from our study match the results of Soltani et al. (91) who showed that weight loss programs with nutritional assistance help adults reduce bone loss while caloric restriction alone harms bone health (92). Our research results on vitamin B supplementation demonstrate that vitamin B intake affects bone mineral protection and fracture susceptibility. The study by Luo et al. (92) discovered that B vitamin supplementation especially folate vitamin B6 and vitamin B12 leads to better bone health and decreased homocysteine-related fracture risk (93). Our research found that homocysteine pathway interventions succeeded in lowering plasma homocysteine levels together with bone resorption indicators which supported the earlier study results of Garcia Lopez et al. and Stone et al. (93, 94) who found that B vitamin supplementation reduced fracture risk among treated populations (92, 94).

The research results show that macro and micronutrient interventions lead to consistent reductions of TNF-*α* and IL-6 and CRP and oxidative damage markers which demonstrate their effects on inflammation and oxidative stress. The findings of this study confirm previous meta-analysis results which showed that antioxidant vitamins and trace elements in musculoskeletal disorders improved inflammatory profiles and reduced oxidative stress (95–97).

The analysis of implant and postoperative results shows that our research produced lower polyethylene wear together with better fracture union rates and improved functional recovery, which confirms earlier research about vitamin E–enhanced polyethylene in hip arthroplasty and vitamin K–calcium combinations that improve site-specific BMD (98–100). The evidence shows that vitamin D treatment for sarcopenia in orthopedic patients has resulted in improvements to both muscle strength and functional performance (101).

The study results demonstrate that targeted macro and micronutrient supplementation improves bone health and lowers fracture probability and controls inflammation and enhances recovery time, which demonstrates the necessity of nutritional treatment together with medication and rehabilitation programs in orthopedic patient treatment.

### Strengths and limitations

#### Strengths

- The analysis included 7 major outcome groups (Bone Health, Bone Turnover, Fracture Healing, Inflammation, Functional Outcomes, Postoperative Recovery, Miscellaneous) which contained more than 15 studies for each major category. The research studied all nutritional and rehabilitation methods which existed at that time.
- The research involved more than 15,000 participants who belonged to different groups which increased both research statistical power and study findings ability to apply to other situations.
- The researchers conducted random-effects meta-analysis through inverse variance weighting which enabled them to compute standardized mean difference (SMD) results while examining different study outcomes.
- The studies were analyzed through Cochrane RoB 2.0 and Jadad scale and GRADE assessment which established high assurance for the evidence quality which supported most study outcomes.
- The researchers conducted publication bias assessment through funnel plots and Egger’s tests which showed that most outcome categories had minimal publication bias except for postoperative and metabolic results.
- The study produced multiple outcomes which had real clinical value. These outcomes measured BMD and fracture healing and biochemical markers and functional scores and pain and short-term metabolic recovery.

#### Limitations

- The presence of non-randomized studies and protocol-based studies in some outcomes raised the total bias risk for specific analyses.
- The studies about postoperative recovery and inflammation showed limited follow-up periods which lasted from one to twelve weeks, which made it impossible to assess their long-term impact.
- The different measurement methods and units and biomarkers used by studies prevented standardization which led to measurement problems that affected SMD results.
- This review included only English-language studies which introduced language bias while restricting the complete evidence base. Relevant studies published in other languages may have been excluded.
- The included studies used brief follow-up periods which lasted between 1 and 12 weeks to study the effects of treatments on bone mineral density and fracture healing and long-term functional restoration.
- The different measurement methods used in studies together with their choice of reporting units and selected biomarkers created standardization problems that resulted in study results showing varying outcomes.
- The use of various outcome measures which included BMD and fracture healing and biochemical markers and functional scores and pain and short-term metabolic recovery led to clinical study heterogeneity which made it impossible to compare studies.

## Conclusion

The findings of this complete meta-analysis prove that orthopedic patients receive important advantages from specific nutritional and rehabilitation treatment methods. The treatment methods resulted in better bone mineral density levels and reduced bone turnover biochemical markers while decreasing both inflammation and oxidative damage. The results showed important improvements in both postoperative recovery and short-term metabolic results. The study found that various results which assessed fracture healing and functional performance and other different aspects showed inconsistent results which failed to achieve consistent significance. The majority of studies included in the research maintained moderate to high quality standards according to Jadad scoring and RoB 2.0 and GRADE assessments while showing minimal signs of publication bias. The studies exhibited substantial heterogeneity because researchers employed different intervention methods and followed various patient groups over distinct time frames. Nutritional interventions show positive effects but the evidence for their impact on postoperative recovery and short-term metabolic results remains uncertain because of publication bias and small-study effects. The research findings demonstrate that customized nutritional and rehabilitation programs serve as effective supplementary treatments which help orthopedic patients achieve better bone health and reduced inflammation and faster recovery. The research results need to be verified through future randomized controlled trials which must be designed as large-scale studies and their outcomes should be used to create standard clinical treatment guidelines.

## Data Availability

The raw data supporting the conclusions of this article will be made available by the authors, without undue reservation.

## References

[1] Wnuk-Scardaccione A Cima MS. Limb Osseointegration—how important is the role of nutrition in the process? Nutrients. (2025) 17:606. doi: 10.3390/nu17040606, 40004935 PMC11858377

[2] Osman A Maya T Doshi N Abbas H Marquez T Hasasna Z . Post-stroke bone fragility: early bone loss, risk factors, and recovery considerations. Cureus. (2025) 17:e89282. doi: 10.7759/cureus.89282, 40904972 PMC12404217

[3] Cianferotti L Bifolco G Caffarelli C Mazziotti G Migliaccio S Napoli N . Nutrition, vitamin D, and calcium in elderly patients before and after a hip fracture and their impact on the musculoskeletal system: a narrative review. Nutrients. (2024) 16:1773. doi: 10.3390/nu16111773, 38892706 PMC11174536

[4] Martiniakova M Babikova M Mondockova V Blahova J Kovacova V Omelka R. The role of macronutrients, micronutrients and flavonoid polyphenols in the prevention and treatment of osteoporosis. Nutrients. (2022) 14:523. doi: 10.3390/nu14030523, 35276879 PMC8839902

[5] Je M Kang K Yoo J-I Kim Y. The influences of macronutrients on bone mineral density, bone turnover markers, and fracture risk in elderly people: a review of human studies. Nutrients. (2023) 15:4386. doi: 10.3390/nu15204386, 37892460 PMC10610213

[6] Li Z Wang X Yu Y Jing Y Du H Liu W . Nutritional alterations, adverse consequences, and comprehensive assessment in spinal cord injury: a review. Front Nutr. (2025) 12:1576976. doi: 10.3389/fnut.2025.1576976, 40416388 PMC12098053

[7] Briguglio M. Integration of Nutritional Support in Orthopedics: Dietary and nutritional Aspects of Surgical Patients (2022)

[8] Marks R. Does nutrition impact hip fragility fracture risk and outcomes? The case of malnutrition and bone health. Mortality. (2022) 15:16.

[9] Katoch S Easwaran R Thacker H Singh AK Moonot P Mukherjee AN . Expert consensus on the role of nutraceuticals in bone, joints, and muscle health. Int J Orthop Sci. (2022) 8:286–92. doi: 10.22271/ortho.2022.v8.i3e.3211

[10] de Sire A Lippi L Aprile V Calafiore D Folli A D’Abrosca F . Pharmacological, nutritional, and rehabilitative interventions to improve the complex management of osteoporosis in patients with chronic obstructive pulmonary disease: a narrative review. Journal of Personalized Medicine. (2022) 12:1626. doi: 10.3390/jpm12101626, 36294765 PMC9604650

[11] Torbergsen AC Watne LO Frihagen F Wyller TB Mowè M. Effects of nutritional intervention upon bone turnover in elderly hip fracture patients. Randomized controlled trial. Clin Nutr ESPEN. (2019) 29:52–8. doi: 10.1016/j.clnesp.2017.11.012, 30661701

[12] Invernizzi M de Sire A D’Andrea F Carrera D Renò F Migliaccio S . Effects of essential amino acid supplementation and rehabilitation on functioning in hip fracture patients: a pilot randomized controlled trial. Aging Clin Exp Res. (2019) 31:1517–24. doi: 10.1007/s40520-018-1090-y, 30539540

[13] Villareal DT Fontana L Weiss EP Racette SB Steger-May K Schechtman KB . Bone mineral density response to caloric restriction–induced weight loss or exercise-induced weight loss: a randomized controlled trial. Arch Intern Med. (2006) 166:2502–10. doi: 10.1001/archinte.166.22.2502 17159017

[14] Ekinci O Yanık S Terzioğlu Bebitoğlu B Yılmaz Akyüz E Dokuyucu A Erdem Ş. Effect of calcium β-hydroxy-β-methylbutyrate (CaHMB), vitamin D, and protein supplementation on postoperative immobilization in malnourished older adult patients with hip fracture: a randomized controlled study. Nutr Clin Pract. (2016) 31:829–35. doi: 10.1177/0884533616629628, 26965178

[15] Kemmler W Kohl M Jakob F Engelke K von Stengel S. Effects of high intensity dynamic resistance exercise and whey protein supplements on osteosarcopenia in older men with low bone and muscle mass. Final results of the randomized controlled FrOST study. Nutrients. (2020) 12:2341. doi: 10.3390/nu12082341, 32764397 PMC7468852

[16] Wyers CE Reijven PL Breedveld-Peters JJ Denissen KF Schotanus MG van Dongen MC . Efficacy of nutritional intervention in elderly after hip fracture: a multicenter randomized controlled trial. J Gerontol A Biol Sci Med Sci. (2018) 73:1429–37. doi: 10.1093/gerona/gly030 30204859 PMC6132112

[17] Burt LA Billington EO Rose MS Raymond DA Hanley DA Boyd SK. Effect of high-dose vitamin D supplementation on volumetric bone density and bone strength: a randomized clinical trial. JAMA. (2019) 322:736–45. doi: 10.1001/jama.2019.11889, 31454046 PMC6714464

[18] Van Erp JH Massier JR Halma JJ Snijders TE De Gast A. 2-year results of an RCT of 2 uncemented isoelastic monoblock acetabular components: lower wear rate with vitamin E blended highly cross-linked polyethylene compared to ultra-high molecular weight polyethylene. Acta Orthop. (2020) 91:254–9. doi: 10.1080/17453674.2020.1730073, 32098534 PMC8023900

[19] Busch A Jäger M Klebingat S Baghdadi J Flörkemeier T Hütter F . Vitamin E-blended highly cross-linked polyethylene liners in total hip arthroplasty: a randomized, multicenter trial using virtual CAD-based wear analysis at 5-year follow-up. Arch Orthop Trauma Surg. (2020) 140:1859–66. doi: 10.1007/s00402-020-03358-x, 32048017

[20] Maria S Swanson MH Enderby LT D'Amico F Enderby B Samsonraj RM . Melatonin-micronutrients osteopenia treatment study (MOTS): a translational study assessing melatonin, strontium (citrate), vitamin D3 and vitamin K2 (MK7) on bone density, bone marker turnover and health related quality of life in postmenopausal osteopenic women following a one-year double-blind RCT and on osteoblast-osteoclast co-cultures. Aging Albany. (2017) 9:256–85. doi: 10.18632/aging.101158 PMC5310667 28130552

[21] Tanaka S Miyazaki T Uemura Y Miyakawa N Gorai I Nakamura T . Comparison of concurrent treatment with vitamin K2 and risedronate compared with treatment with risedronate alone in patients with osteoporosis: Japanese osteoporosis intervention Trial-03. J Bone Miner Metab. (2017) 35:385–95. doi: 10.1007/s00774-016-0768-5, 27484436

[22] Salovaara K Tuppurainen M Kärkkäinen M Rikkonen T Sandini L Sirola J . Effect of vitamin D3 and calcium on fracture risk in 65-to 71-year-old women: a population-based 3-year randomized, controlled trial—the OSTPRE-FPS. J Bone Miner Res. (2010) 25:1487–95. doi: 10.1002/jbmr.48, 20200964

[23] Govender S Csimma C Genant HK Valentin-Opran A Amit Y Arbel R . Recombinant human bone morphogenetic protein-2 for treatment of open tibial fractures: a prospective, controlled, randomized study of four hundred and fifty patients. J Bone Joint Surg. (2002) 84:2123–34. doi: 10.2106/00004623-200212000-00001, 12473698

[24] Friedlaender GE Perry CR Cole JD Cook SD Cierny G Muschler GF . Osteogenic protein-1 (bone morphogenetic protein-7) in the treatment of tibial nonunions: a prospective, randomized clinical trial comparing rhOP-1 with fresh bone autograft. JBJS. (2001) 83:S151–8. PMC1425155 11314793

[25] Ekrol I Duckworth AD Ralston SH McQueen MM. The influence of vitamin C on the outcome of distal radial fractures: a double-blind, randomized controlled trial. J Bone Joint Surg. (2014) 96:1451–9. doi: 10.2106/JBJS.M.00268, 25187584

[26] Athinarayanan SJ Adams RN Hallberg SJ McKenzie AL Bhanpuri NH Campbell WW . Long-term effects of a novel continuous remote care intervention including nutritional ketosis for the management of type 2 diabetes: a 2-year non-randomized clinical trial. Front Endocrinol. (2019) 10:450805. doi: 10.3389/fendo.2019.00348, 31231311 PMC6561315

[27] Barnosky A Kroeger CM Trepanowski JF Klempel MC Bhutani S Hoddy KK . Effect of alternate day fasting on markers of bone metabolism: an exploratory analysis of a 6-month randomized controlled trial. Nutr Healthy Aging. (2017) 4:255–63. doi: 10.3233/NHA-170031, 29276795 PMC5734119

[28] Schnyder G Roffi M Flammer Y Pin R Hess OM. Effect of homocysteine-lowering therapy with folic acid, vitamin B12, and vitamin B6 on clinical outcome after percutaneous coronary intervention: the Swiss heart study: a randomized controlled trial. JAMA. (2002) 288:973–9. doi: 10.1001/jama.288.8.973 12190367

[29] Bischoff-Ferrari HA Dawson-Hughes B Orav EJ Staehelin HB Meyer OW Theiler R . Monthly high-dose vitamin D treatment for the prevention of functional decline: a randomized clinical trial. JAMA Intern Med. (2016) 176:175–83. doi: 10.1001/jamainternmed.2015.7148, 26747333

[30] Maier K Selig M Haddouche A Haunschild M Hauschild O Khalili I . Vitamin E-enriched medium cross-linked polyethylene in total knee arthroplasty (VIKEP): clinical outcome, oxidation profile, and wear analysis in comparison to standard polyethylene—study protocol for a randomized controlled trial. Trials. (2024) 25:27. doi: 10.1186/s13063-023-07811-1, 38183062 PMC10768156

[31] Torbergsen AC Watne LO Wyller TB Frihagen F Strømsøe K Bøhmer T . Micronutrients and the risk of hip fracture: case–control study. Clin Nutr. (2017) 36:438–43. doi: 10.1016/j.clnu.2015.12.014, 26795217

[32] Ong MT-y Lu X Choi BC-y Wan S-W Wang Q Man GC-w . Vitamin D as an intervention for improving quadriceps muscle strength in patients after anterior cruciate ligament reconstruction: study protocol for a randomized double-blinded, placebo-controlled clinical trial. Trials. (2024) 25:251. doi: 10.1186/s13063-024-08094-w 38605374 PMC11008016

[33] Li W Chen M Chen F Li Y Zhong Y Lu Y . Vitamin D combined with whole-body vibration training for the treatment of osteo-sarcopenia: study protocol for a randomized controlled trial. Trials. (2024) 25:638. doi: 10.1186/s13063-024-08498-8, 39350307 PMC11440726

[34] Cohen TR Hazell TJ Vanstone CA Plourde H Rodd CJ Weiler HA. A family-centered lifestyle intervention to improve body composition and bone mass in overweight and obese children 6 through 8 years: a randomized controlled trial study protocol. BMC Public Health. (2013) 13:383. doi: 10.1186/1471-2458-13-383 23617621 PMC3648398

[35] Plante L. Effect of High-Dose Vitamin D Supplementation on Bone Density in Youth with Osteogenesis Imperfecta: A Randomized Controlled Trial. Montreal: McGill University (2015). 10.1016/j.bone.2016.02.013 26924265

[36] Moppett IK Greenhaff PL Ollivere BJ Joachim T Lobo DN Rowlands M. Pre-operative nutrition in neck of femur trial (POINT)-carbohydrate loading in patients with fragility hip fracture: study protocol for a randomised controlled trial. Trials. (2014) 15:475. doi: 10.1186/1745-6215-15-475, 25472724 PMC4289274

[37] Plante L Veilleux L-N Glorieux FH Weiler H Rauch F. Effect of high-dose vitamin D supplementation on bone density in youth with osteogenesis imperfecta: a randomized controlled trial. Bone. (2016) 86:36–42. doi: 10.1016/j.bone.2016.02.013, 26924265

[38] Hansen KE Johnson RE Chambers KR Johnson MG Lemon CC Vo TNT . Treatment of vitamin D insufficiency in postmenopausal women: a randomized clinical trial. JAMA Intern Med. (2015) 175:1612–21. doi: 10.1001/jamainternmed.2015.3874, 26237520 PMC4594209

[39] Kärkkäinen M Tuppurainen M Salovaara K Sandini L Rikkonen T Sirola J . Effect of calcium and vitamin D supplementation on bone mineral density in women aged 65–71 years: a 3-year randomized population-based trial (OSTPRE-FPS). Osteoporos Int. (2010) 21:2047–55. doi: 10.1007/s00198-009-1167-8, 20204604

[40] Reid IR Ames R Mason B Reid HE Bacon CJ Bolland MJ . Randomized controlled trial of calcium supplementation in healthy, nonosteoporotic, older men. Arch Intern Med. (2008) 168:2276–82. doi: 10.1001/archinte.168.20.2276, 19001206

[41] Walsh JS Jacques RM Schomburg L Hill TR Mathers JC Williams GR . Effect of selenium supplementation on musculoskeletal health in older women: a randomised, double-blind, placebo-controlled trial. Lancet Healthy Longev. (2021) 2:e212–21. doi: 10.1016/s2666-7568(21)00051-9 33842907 PMC8020713

[42] König D Oesser S Scharla S Zdzieblik D Gollhofer A. Specific collagen peptides improve bone mineral density and bone markers in postmenopausal women—a randomized controlled study. Nutrients. (2018) 10:97. doi: 10.3390/nu10010097, 29337906 PMC5793325

[43] van der Veen HC van den Akker-Scheek I Bulstra SK van Raay JJ. Wear, bone density, functional outcome and survival in vitamin E-incorporated polyethylene cups in reversed hybrid total hip arthroplasty: design of a randomized controlled trial. BMC Musculoskelet Disord. (2012) 13:178. doi: 10.1186/1471-2474-13-178, 22994935 PMC3517763

[44] Sotome S Ae K Okawa A Ishizuki M Morioka H Matsumoto S . Efficacy and safety of porous hydroxyapatite/type 1 collagen composite implantation for bone regeneration: a randomized controlled study. J Orthop Sci. (2016) 21:373–80. doi: 10.1016/j.jos.2016.01.007, 26961287

[45] Herrmann M Umanskaya N Traber L Schmidt-Gayk H Menke W Lanzer G . The effect of B-vitamins on biochemical bone turnover markers and bone mineral density in osteoporotic patients: a 1-year double blind placebo controlled trial. Clin Chem Lab Med. (2007) 45:1785–92. doi: 10.1515/CCLM.2007.352 18020969

[46] Spector TD Calomme MR Anderson SH Clement G Bevan L Demeester N . Choline-stabilized orthosilicic acid supplementation as an adjunct to calcium/vitamin D3 stimulates markers of bone formation in osteopenic females: a randomized, placebo-controlled trial. BMC Musculoskelet Disord. (2008) 9:85. doi: 10.1186/1471-2474-9-85, 18547426 PMC2442067

[47] Toole JF Malinow MR Chambless LE Spence JD Pettigrew LC Howard VJ . Lowering homocysteine in patients with ischemic stroke to prevent recurrent stroke, myocardial infarction, and death: the vitamin intervention for stroke prevention (VISP) randomized controlled trial. JAMA. (2004) 291:565–75. doi: 10.1001/jama.291.5.565, 14762035

[48] Loftis JM Ast HK Bruton AM Srikanth P Ramesh R Erikson DW . Multinutrient supplementation in children with ADHD reduced pro-and anti-inflammatory immune factors in the MADDY randomized controlled trial. J Atten Disord. (2025). doi: 10.1177/10870547251397701 PMC12959162 41571599

[49] Ramón R Holguín E Chiriboga JD Rubio N Ballesteros C Ezechieli M. Anti-inflammatory effect of vitamin C during the postoperative period in patients subjected to total knee arthroplasty: a randomized controlled trial. J Pers Med. (2023) 13:1299. doi: 10.3390/jpm13091299, 37763067 PMC10532858

[50] Abdoulhossein D Taheri I ali Saba M Akbari H Shafagh S Zataollah A. Effect of vitamin C and vitamin E on lung contusion: a randomized clinical trial study. Ann Med Surg. (2018) 36:152–7. doi: 10.1016/j.amsu.2018.10.026, 30479762 PMC6240669

[51] Zamani B Taghvaee F Akbari H Mohtashamian A Sharifi N. Effects of selenium supplementation on the indices of disease activity, inflammation and oxidative stress in patients with rheumatoid arthritis: a randomized clinical trial. Biol Trace Elem Res. (2024) 202:1457–67. doi: 10.1007/s12011-023-03782-1, 37477848

[52] Alehagen U Alexander J Aaseth J Larsson A. Decrease in inflammatory biomarker concentration by intervention with selenium and coenzyme Q10: a subanalysis of osteopontin, osteoprotergerin, TNFr1, TNFr2 and TWEAK. J Inflamm. (2019) 16:5. doi: 10.1186/s12950-019-0210-6, 30923464 PMC6421641

[53] Sedighinejad A Imantalab V Mirmansouri A Jouryabi AM Kanani G Sheikhani NN . Effects of low-dose selenium on the inflammatory response in coronary artery bypass graft surgery: a clinical trial. Iran Red Crescent Med J. (2016) 18:e37918. doi: 10.5812/ircmj.37918, 27795837 PMC5070486

[54] Tantavisut S Tanavalee A Honsawek S Suantawee T Ngarmukos S Adisakwatana S . Effect of vitamin E on oxidative stress level in blood, synovial fluid, and synovial tissue in severe knee osteoarthritis: a randomized controlled study. BMC Musculoskelet Disord. (2017) 18:281. doi: 10.1186/s12891-017-1637-7, 28662656 PMC5492918

[55] Huang H-Y Helzlsouer KJ Appel LJ. The effects of vitamin C and vitamin E on oxidative DNA damage: results from a randomized controlled trial. Cancer Epidemiol Biomarkers Prev. (2000) 9:647–52. 10919732

[56] Lassig AAD Wilson AC Jungbauer WN Joseph AM Lindgren B Odland R. The effects of supplemental vitamin c in mandibular fracture patients: a randomized clinical trial. Recent Prog Nutr. (2023) 3:1–17.

[57] Gunton JE Girgis CM Lau T Vicaretti M Begg L Flood V. Vitamin C improves healing of foot ulcers: a randomised, double-blind, placebo-controlled trial. Br J Nutr. (2021) 126:1451–8. doi: 10.1017/S0007114520003815, 32981536

[58] Blass SC Goost H Tolba RH Stoffel-Wagner B Kabir K Burger C . Time to wound closure in trauma patients with disorders in wound healing is shortened by supplements containing antioxidant micronutrients and glutamine: a PRCT. Clin Nutr. (2012) 31:469–75. doi: 10.1016/j.clnu.2012.01.002, 22284340

[59] Kawaguchi H Oka H Jingushi S Izumi T Fukunaga M Sato K . A local application of recombinant human fibroblast growth factor 2 for tibial shaft fractures: a randomized, placebo-controlled trial. J Bone Miner Res. (2010) 25:2735–43. doi: 10.1002/jbmr.146, 20533373

[60] Daniels TR Younger AS Penner MJ Wing KJ Le IL Russell IS . Prospective randomized controlled trial of hindfoot and ankle fusions treated with rhPDGF-BB in combination with a β-TCP-collagen matrix. Foot Ankle Int. (2015) 36:739–48. doi: 10.1177/1071100715576370 25848134

[61] Calori G Tagliabue L Gala L d’Imporzano M Peretti G Albisetti W. Application of rhBMP-7 and platelet-rich plasma in the treatment of long bone non-unions: a prospective randomised clinical study on 120 patients. Injury. (2008) 39:1391–402. doi: 10.1016/j.injury.2008.08.011 19027898

[62] Scemama C Anract P Dumaine V Babinet A Courpied JP Hamadouche M. Does vitamin E-blended polyethylene reduce wear in primary total hip arthroplasty: a blinded randomised clinical trial. Int Orthop. (2017) 41:1113–8. doi: 10.1007/s00264-016-3320-2 27815591

[63] Rochcongar G Buia G Bourroux E Dunet J Chapus V Hulet C. Creep and wear in vitamin E-infused highly cross-linked polyethylene cups for total hip arthroplasty: a prospective randomized controlled trial. JBJS. (2018) 100:107–14. doi: 10.2106/JBJS.16.01379, 29342060

[64] Salemyr M Muren O Ahl T Bodén H Chammout G Stark A . Vitamin-E diffused highly cross-linked polyethylene liner compared to standard liners in total hip arthroplasty. A randomized, controlled trial. Int Orthop. (2015) 39:1499–505. doi: 10.1007/s00264-015-2680-3, 25631687

[65] Kjærgaard K Ding M Jensen C Bragdon C Malchau H Andreasen CM . Vitamin E-doped total hip arthroplasty liners show similar head penetration to highly cross-linked polyethylene at five years: a multi-arm randomized controlled trial. Bone Joint J. (2020) 102-B:1303–10. doi: 10.1302/0301-620X.102B10.BJJ-2020-0138.R1, 32993343 PMC7517722

[66] Rønn SH Harsløf T Pedersen SB Langdahl BL. Vitamin K2 (menaquinone-7) prevents age-related deterioration of trabecular bone microarchitecture at the tibia in postmenopausal women. Eur J Endocrinol. (2016) 175:541–9. doi: 10.1530/eje-16-0498 27625301

[67] Tajari F Ghamari BT Kafiabadi MJ Shariatzade H Biglari F Nasab OM . Effect of vitamin C injection on flexor tendon healing in zone II: a randomized controlled trial. Cureus. (2026) 18:e102075. doi: 10.7759/cureus.102075 41732638 PMC12925623

[68] Mohammadivahedi F Sadeghifar A Farsinejad A Jambarsang S Mirhosseini H. Comparative efficacy of platelet-rich plasma (PRP) injection versus PRP combined with vitamin C injection for partial-thickness rotator cuff tears: a randomized controlled trial. J Orthop Surg Res. (2024) 19:426. doi: 10.1186/s13018-024-04917-3, 39044241 PMC11267806

[69] Houston DK Marsh AP Neiberg RH Demons JL Campos CL Kritchevsky SB . Vitamin D supplementation and muscle power, strength and physical performance in older adults: a randomized controlled trial. Am J Clin Nutr. (2023) 117:1086–95. doi: 10.1016/j.ajcnut.2023.04.021, 37084814 PMC10447505

[70] Zhu K Austin N Devine A Bruce D Prince RL. A randomized controlled trial of the effects of vitamin D on muscle strength and mobility in older women with vitamin D insufficiency. J Am Geriatr Soc. (2010) 58:2063–8. doi: 10.1111/j.1532-5415.2010.03142.x, 21054285

[71] Agergaard J Trøstrup J Uth J Iversen JV Boesen A Andersen JL . Does vitamin-D intake during resistance training improve the skeletal muscle hypertrophic and strength response in young and elderly men?–a randomized controlled trial. Nutr Metabol. (2015) 12:32. doi: 10.1186/s12986-015-0029-y, 26430465 PMC4589960

[72] Jendricke P Centner C Zdzieblik D Gollhofer A König D. Specific collagen peptides in combination with resistance training improve body composition and regional muscle strength in premenopausal women: a randomized controlled trial. Nutrients. (2019) 11:892. doi: 10.3390/nu11040892, 31010031 PMC6521629

[73] Songpatanasilp T Chailurkit L-o Nichachotsalid A Chantarasorn M. Combination of alfacalcidol with calcium can improve quadriceps muscle strength in elderly ambulatory Thai women who have hypovitaminosis D: a randomized controlled trial. J Med Assoc Thail. (2011) 92:30. 19894330

[74] Jiang W Xu H Jiang X Zhan Y Ju Y Xie J . Efficacy of vitamin C as glucocorticoid substitute for reducing pain and inflammation after total hip arthroplasty: a randomized controlled trial. J Bone Joint Surg. (2025) 107:1123–33. doi: 10.2106/JBJS.24.01080, 40208930

[75] Yarahmadi A Saeed Modaghegh M-H Mostafavi-Pour Z Azarpira N Mousavian A Bonakdaran S . The effect of platelet-rich plasma-fibrin glue dressing in combination with oral vitamin E and C for treatment of non-healing diabetic foot ulcers: a randomized, double-blind, parallel-group, clinical trial. Expert Opin Biol Ther. (2021) 21:687–96. doi: 10.1080/14712598.2021.1897100, 33646060

[76] Chaudhary NK Sunuwar DR Sharma R Karki M Timilsena MN Gurung A . The effect of pre-operative carbohydrate loading in femur fracture: a randomized controlled trial. BMC Musculoskelet Disord. (2022) 23:819. doi: 10.1186/s12891-022-05766-z, 36042436 PMC9424836

[77] Lai Y Cai Y Ding Z Huang C Luo Z Zhou Z. Effect of preoperative carbohydrate loading on postoperative recovery of individuals who have type 2 diabetes after total knee arthroplasty: a randomized controlled trial. J Arthroplast. (2025) 40:665–71. doi: 10.1016/j.arth.2024.09.016, 39293701

[78] Kumar M Patil NS Mohapatra N Yadav A Sindwani G Dhingra U . Preoperative carbohydrate loading reduces perioperative insulin resistance and hastens functional recovery of remnant liver after living donor hepatectomy: an open-label randomized controlled trial. Hepatol Int. (2025) 19:1151–61. doi: 10.1007/s12072-025-10831-5, 40389625

[79] Liu B Wang Y Liu S Zhao T Zhao B Jiang X . A randomized controlled study of preoperative oral carbohydrate loading versus fasting in patients undergoing elective craniotomy. Clin Nutr. (2019) 38:2106–12. doi: 10.1016/j.clnu.2018.11.008, 30497695

[80] Walnum CF Straszek M Husted K Bagger J Fahrenholtz IL Melin A . Post-exercise carbohydrate intake in the early postoperative phase following primary total hip and knee arthroplasties–a randomized controlled trial. J Phys Med Rehabil. (2023) 5:28–33. doi: 10.33696/rehabilitation.5.037

[81] Bousquet-Dion G Awasthi R Loiselle S-È Minnella EM Agnihotram RV Bergdahl A . Evaluation of supervised multimodal prehabilitation programme in cancer patients undergoing colorectal resection: a randomized control trial. Acta Oncol. (2018) 57:849–59. doi: 10.1080/0284186X.2017.1423180, 29327644

[82] Vallibhakara SA-O Nakpalat K Sophonsritsuk A Tantitham C Vallibhakara O. Effect of vitamin E supplement on bone turnover markers in postmenopausal osteopenic women: a double-blind, randomized, placebo-controlled trial. Nutrients. (2021) 13:4226. doi: 10.3390/nu13124226, 34959779 PMC8709036

[83] Clifford T Ventress M Allerton DM Stansfield S Tang JC Fraser WD . The effects of collagen peptides on muscle damage, inflammation and bone turnover following exercise: a randomized, controlled trial. Amino Acids. (2019) 51:691–704. doi: 10.1007/s00726-019-02706-5, 30783776

[84] Jarusriwanna A Phusunti S Chotiyarnwong P Unnanuntana A. High-dose versus low-dose ergocalciferol for correcting hypovitaminosis D after fragility hip fracture: a randomized controlled trial. BMC Geriatr. (2021) 21:72. doi: 10.1186/s12877-021-02023-1, 33478397 PMC7818778

[85] Biboulet P Bringuier S Smilevitch P Loupec T Thuile C Pencole M . Preoperative epoetin-α with intravenous or oral iron for major orthopedic surgery: a randomized controlled trial. Anesthesiology. (2018) 129:710–20. doi: 10.1097/ALN.0000000000002376 30074935

[86] Peterson DF McKibben NS Hutchison CE Lancaster K Yang CJ Dekeyser GJ . Role of single-dose intravenous iron therapy for the treatment of anaemia after orthopaedic trauma: protocol for a pilot randomised controlled trial. BMJ Open. (2023) 13:e069070. doi: 10.1136/bmjopen-2022-069070, 36944463 PMC10032390

[87] Feagan BG Wong CJ Kirkley A Johnston D Smith FC Whitsitt P . Erythropoietin with iron supplementation to prevent allogeneic blood transfusion in total hip joint arthroplasty: a randomized, controlled trial. Ann Intern Med. (2000) 133:845–54. 11103054 10.7326/0003-4819-133-11-200012050-00008

[88] Lustberg MB Orchard TS Reinbolt R Andridge R Pan X Belury M . Randomized placebo-controlled pilot trial of omega 3 fatty acids for prevention of aromatase inhibitor-induced musculoskeletal pain. Breast Cancer Res Treat. (2018) 167:709–18. doi: 10.1007/s10549-017-4559-z, 29101597 PMC5809189

[89] MacFarlane LA Cook NR Kim E Lee IM Iversen MD Gordon D . The effects of vitamin D and marine omega-3 fatty acid supplementation on chronic knee pain in older US adults: results from a randomized trial. Arthritis Rheumatol. (2020) 72:1836–44. doi: 10.1002/art.41416 32583982 PMC7874905

[90] López-Vidriero E Olivé-Vilas R López-Capapé D Varela-Sende L López-Vidriero R Til-Pérez L. Efficacy and tolerability of progen, a nutritional supplement based on innovative plasma proteins, in ACL reconstruction: a multicenter randomized controlled trial. Orthop J Sports Med. (2019) 7:2325967119827237. doi: 10.1177/2325967119827237, 30834280 PMC6393838

[91] Soltani S Hunter G Kazemi A Shab-Bidar S. The effects of weight loss approaches on bone mineral density in adults: a systematic review and meta-analysis of randomized controlled trials. Osteoporos Int. (2016) 27:2655–71. doi: 10.1007/s00198-016-3617-4 27154437

[92] Luo Y Zheng S Jiang S Yang G Pavel V Ji H . B vitamins and bone health: a meta-analysis with trial sequential analysis of randomized controlled trials. Osteoporos Int. (2024) 35:1645–59. doi: 10.1007/s00198-024-07150-0 38953947

[93] Garcia Lopez M Baron JA Omsland TK Søgaard AJ Meyer HE. Homocysteine lowering treatment and the risk of fracture: secondary analysis of a randomized controlled trial and an updated Meta analysis. J Bone Miner Res Plus. (2018) 2:295–303. doi: 10.1002/jbm4.10045, 30283911 PMC6139704

[94] Stone KL Lui LY Christen WG Troen AM Bauer DC Kado D . Effect of combination folic acid, vitamin B6, and vitamin B12 supplementation on fracture risk in women: a randomized, controlled trial. J Bone Miner Res. (2017) 32:2331–8. doi: 10.1002/jbmr.3229, 29244251 PMC5734110

[95] Alqurayqiri ZM Alharbi TM. Efficacy and safety of combining NSAIDs with vitamin B for musculoskeletal pain: a systematic review and meta-analysis. Saudi J Emerg Med. (2025) 6:52–64. doi: 10.24911/SJEMed.72-1725634958

[96] Canter P Wider B Ernst E. The antioxidant vitamins a, C, E and selenium in the treatment of arthritis: a systematic review of randomized clinical trials. Rheumatology. (2007) 46:1223–33. doi: 10.1093/rheumatology/kem116, 17522095

[97] Hung K-C Chiang M-H Wu S-C Chang Y-J Ho C-N Wang L-K . A meta-analysis of randomized clinical trials on the impact of oral vitamin C supplementation on first-year outcomes in orthopedic patients. Sci Rep. (2021) 11:9225. doi: 10.1038/s41598-021-88864-7, 33927326 PMC8085077

[98] Li Z Xiang S Wu C Wang Y Weng X. Vitamin E highly cross-linked polyethylene reduces mid-term wear in primary total hip replacement: a meta-analysis and systematic review of randomized clinical trials using radiostereometric analysis. EFORT Open Rev. (2021) 6:759–70. doi: 10.1302/2058-5241.6.200072, 34667647 PMC8489480

[99] Hu L Ji J Li D Meng J Yu B. The combined effect of vitamin K and calcium on bone mineral density in humans: a meta-analysis of randomized controlled trials. J Orthop Surg Res. (2021) 16:592. doi: 10.1186/s13018-021-02728-4, 34649591 PMC8515712

[100] Xie C Gong J Zheng C Zhang J Gao J Tian C . Effects of vitamin K supplementation on bone mineral density at different sites and bone metabolism in the middle-aged and elderly population: a meta-analysis and systematic review of randomized controlled trials. Bone Joint Res. (2024) 13:750–63. doi: 10.1302/2046-3758.1312.BJR-2024-0053.R1 39657786 PMC11631259

[101] Cheng S-H Chen K-H Chen C Chu W-C Kang Y-N. The optimal strategy of vitamin D for sarcopenia: a network meta-analysis of randomized controlled trials. Nutrients. (2021) 13:3589. doi: 10.3390/nu13103589, 34684590 PMC8541573

[102] Bergvinsson H Zampelis V Sundberg M Tjörnstrand J Flivik G. Vitamin E infused highly cross-linked cemented cups in total hip arthroplasty show good wear pattern and stabilize satisfactorily: a randomized, controlled RSA trial with 5-year follow-up. Acta Orthop. (2022) 93:249. doi: 10.2340/17453674.2022.1517, 35048993 PMC8788680

[103] Bischoff HA Stähelin HB Dick W Akos R Knecht M Salis C . Effects of vitamin D and calcium supplementation on falls: a randomized controlled trial. J Bone Miner Res. (2003) 18:343–51. doi: 10.1359/jbmr.2003.18.2.343, 12568412

[104] Rousseau A-F Foidart-Desalle M Ledoux D Remy C Croisier J-L Damas P . Effects of cholecalciferol supplementation and optimized calcium intakes on vitamin D status, muscle strength and bone health: a one-year pilot randomized controlled trial in adults with severe burns. Burns. (2015) 41:317–25. doi: 10.1016/j.burns.2014.07.005, 25239849

[105] Hedström M Sjöberg H Dalén N Sjöberg K Brosjö E. Positive effects of anabolic steroids, vitamin D and calcium on muscle mass, bone mineral density and clinical function after a hip fracture: a randomised study of 63 women. J Bone Joint Surg Br Vol. (2002) 84-B:497–503. doi: 10.1302/0301-620X.84B4.0840497, 12043767

[106] Jin X Jones G Cicuttini F Wluka A Zhu Z Han W . Effect of vitamin D supplementation on tibial cartilage volume and knee pain among patients with symptomatic knee osteoarthritis: a randomized clinical trial. JAMA. (2016) 315:1005–13. doi: 10.1001/jama.2016.1961, 26954409

[107] Cao Y Jones G Cicuttini F Winzenberg T Wluka A Sharman J . Vitamin D supplementation in the management of knee osteoarthritis: study protocol for a randomized controlled trial. Trials. (2012) 13:131. doi: 10.1186/1745-6215-13-131, 22867111 PMC3503652

[108] Uusi-Rasi K Kannus P Karinkanta S Pasanen M Patil R Lamberg-Allardt C . Study protocol for prevention of falls: a randomized controlled trial of effects of vitamin D and exercise on falls prevention. BMC Geriatr. (2012) 12:12. doi: 10.1186/1471-2318-12-12, 22448872 PMC3342151

[109] He Y Tang X Ning N Chen J Li P Kang P. Effects of preoperative oral electrolyte-carbohydrate nutrition supplement on postoperative outcomes in elderly patients receiving total knee arthroplasty: a prospective randomized controlled trial. Orthop Surg. (2022) 14:2535–44. doi: 10.1111/os.13424, 36040184 PMC9531096

[110] Kadado A Shaw JH Ayoola AS Akioyamen NO North WT Charters MA. Effects of preoperative carbohydrate-rich drinks on immediate postoperative outcomes in total knee arthroplasty: a randomized controlled trial. J Am Acad Orthop Surg. (2022) 30:e833–41. doi: 10.5435/jaaos-d-21-00960 35312650

[111] Miller MD Crotty M Whitehead C Bannerman E Daniels LA. Nutritional supplementation and resistance training in nutritionally at risk older adults following lower limb fracture: a randomized controlled trial. Clin Rehabil. (2006) 20:311–23. doi: 10.1191/0269215506cr942oa, 16719029

[112] Ertural F Çelik GK Özçelik H. Effect of oral carbohydrate solution administered before hip arthroplasty on preoperative anxiety and postoperative patient comfort: a randomized controlled trial. J Perianesth Nurs. (2023) 38:461–8. doi: 10.1016/j.jopan.2022.08.012 36803737

[113] Akbuğa GA Başer M. Effect of preoperative oral liquid carbohydrate intake on blood glucose, fasting-thirst, and fatigue levels: a randomized controlled study. Brazilian J Anesthesiol. (2021) 71:247–53. doi: 10.1016/j.bjane.2021.02.053 PMC9373098 33845098

[114] Liu N Jin Y Wang X Xiang Z Zhang L Feng S. Safety and feasibility of oral carbohydrate consumption before cesarean delivery on patients with gestational diabetes mellitus: a parallel, randomized controlled trial. J Obstet Gynaecol Res. (2021) 47:1272–80. doi: 10.1111/jog.14653, 33403738

